# Heat stress-induced transposon activation correlates with 3D chromatin organization rearrangement in *Arabidopsis*

**DOI:** 10.1038/s41467-020-15809-5

**Published:** 2020-04-20

**Authors:** Linhua Sun, Yuqing Jing, Xinyu Liu, Qi Li, Zhihui Xue, Zhukuan Cheng, Daowen Wang, Hang He, Weiqiang Qian

**Affiliations:** 1 State Key Laboratory of Protein and Plant Gene Research, School of Life Sciences, Peking University, Beijing, 100871 China; 2 Academy for Advanced Interdisciplinary Studies and Peking-Tsinghua Center for Life Sciences, Peking University, Beijing, 100871 China; 3 State Key Laboratory of Plant Genomics and Center for Plant Gene Research, Institute of Genetics and Developmental Biology, Chinese Academy of Sciences, Beijing, 100101 China; 4 College of Agronomy, State Key Laboratory of Wheat and Maize Crop Science, Henan Agricultural University, Longzi Lake Campus, Zhengzhou, 450046 China

**Keywords:** Gene silencing, Chromatin structure, Heat

## Abstract

In higher eukaryotes, heterochromatin is mainly composed of transposable elements (TEs) silenced by epigenetic mechanisms. But, the silencing of certain heterochromatin-associated TEs is disrupted by heat stress. By comparing genome-wide high-resolution chromatin packing patterns under normal or heat conditions obtained through Hi-C analysis, we show here that heat stress causes global rearrangement of the 3D genome in *Arabidopsis thaliana*. Contacts between pericentromeric regions and distal chromosome arms, as well as proximal intra-chromosomal interactions along the chromosomes, are enhanced. However, interactions within pericentromeres and those between distal intra-chromosomal regions are decreased. Many inter-chromosomal interactions, including those within the *KNOT*, are also reduced. Furthermore, heat activation of TEs exhibits a high correlation with the reduction of chromosomal interactions involving pericentromeres, the *KNOT*, the knob, and the upstream and downstream flanking regions of the activated TEs. Together, our results provide insights into the relationship between TE activation and 3D genome reorganization.

## Abstract (web summary)

Heat stress can activate heterochromatin-associated transposon elements (TEs). Here, the authors show that heat stress leads to global rearrangement of 3D genome and TEs activation closely correlates with 3D chromatin organization rearrangement in *Arabidopsis*.

## Introduction

The three-dimensional (3D) configuration of genome is dynamic and can regulate gene expression. Recently, Hi-C technology^1^, which couples proximity ligation-based chromosome conformation capture (3C)^2^, and next generation sequencing has revealed multifaceted and hierarchical 3D genome organization in microbes, animals, and plants (reviewed in ref. ^3^). The *Arabidopsis* chromosomes, like mammalian chromosomes^1^, are organized into A and B compartments^3,4^. Notably, genome annotation shows that these two chromatin compartments are largely in accordance with the euchromatin/heterochromatin landscapes in *Arabidopsis*^4^.

Both Hi-C and fluorescence in situ hybridization (FISH) studies have identified a chromatin structure, the *KNOT*, in *Arabidopsis*^4,5^. The *KNOT* is formed by ten *KNOT* engaged elements (KEEs), or interactive heterochromatic islands, which are similar in interaction patterns to Piwi-interacting RNA clusters, the major controllers of transposable element (TE) silencing in *Drosophila*^4–6^. KEEs have either short- or long-range intrachromosomal and interchromosomal interactions^4,5^ and can also interact with telomeres^4,5^. Intriguingly, KEEs in euchromatic arms are enriched with the repressive histone mark H3K27me1 and small RNAs for gene silencing. TEs are preferentially integrated into KEEs, suggesting a role of the *KNOT* in TE defense^4^. A recent study indicates that *KNOT* can interact with invasive DNA elements, such as transgenes, and the interaction frequency is positively correlated with transgene silencing; this noncanonical silencing mechanism has been designated as *KNOT*-linked silencing^7^.

The knob, first discovered in maize^8^, is another conspicuous chromatin structure in *Arabidopsis*^9^. The knob *hk4s* in *Arabidopsis*, which is cytogenetically discernable, is about hundreds of kilobases close to the pericentromere on the left (top/short) arm of chromosome 4^9,10^. It consists of a low density of expressed genes but a high density of repeats derived from pericentromeric regions^10^. 4C (circular chromosome conformation capture) and Hi-C studies reveal that the knob contains high-frequency intra-knob interactions that interact strongly with pericentromeric regions rather than with nearby euchromatic regions^5,11^. The fact that euchromatic regions do not participate in those strong interactions indicates that there is a mid-range chromosome loop connecting the knob to pericentromeric regions^5,11^.

A large fraction of the eukaryotic genomes is made up of TEs, which can cause mutations and genetic variations by changing their locations within a genome. TEs are usually silenced by DNA methylation, repressive histone modifications, and heterochromatin formation. Under prolonged heat stress, likely because heterochromatin-embedded genes important for heat response and heat tolerance need to be activated, a panel of TEs are transcriptionally activated^12–14^. Unlike TE activation under other conditions, TE activation under heat stress is not related to changes in DNA methylation, histone methylation, and histone acetylation. Changes in MOM1, a chromatin remodeler, and MORC6, a protein involved in transcriptional gene silencing and chromatin condensation, are also unnecessary for heat-induced TE activation^12–15^. However, *Arabidopsis* HIT4, a plant-specific regulator that translocates from the chromocenter to the nucleolus under heat stress^16^, is required for heat-induced TE activation^17^. A mediator subunit, MED14, and another transcription factor IIH subunit, UVH6, are also required for transcriptional activation of TEs under heat stress^15^. For the *ONSEN* family TEs, the presence of heat-responsive elements and binding of heat-responsive elements by heat shock factor A 2 are required for their activation^18^. After heat stress, the activated TEs are silenced and the CAF-1 complex contributes to the resilencing of TEs during the recovery process^13^.

Here we explore the activation of *Arabidopsis* TEs in response to heat and resilencing of TEs during the recovery phase on a genome-wide scale. We unveil a strong correlation between chromatin decondensation and TE activation by heat through precisely mapping the changes of 3D chromatin organization using Hi-C analysis.

## Results

### Heat induces TE activation

**Fig. 1:**
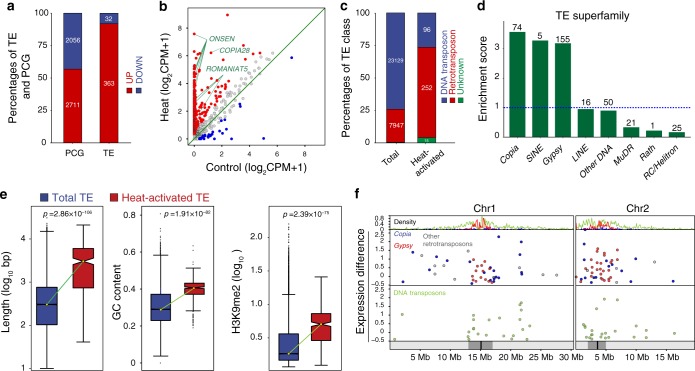
Heat induces TE activation. **a** Percentages of up- and downregulated protein coding genes (PCGs) and transposable elements (TEs) under heat stress. Red bars: upregulation; Blue bars: downregulation. Numbers on the bars indicate the absolute number of TEs and PCGs. **b** Scatter plot of TE CPMs in heat vs. control conditions. CPM counts per million. Red dots: upregulated TEs under heat stress. Blue dots: downregulated TEs under heat stress. **c** The percentages of DNA transposons and retrotransposons for all (total) and for heat-activated TEs. Numbers marked on the bars indicate the absolute numbers of heat-activated TEs and total TEs from different TE classes. **d** The enrichment scores of TE superfamilies of heat-activated TEs. Each enrichment score was calculated as the ratio of the percentage of a certain TE superfamily in heat-activated TEs to that in all TEs. Numbers above the bars indicate the absolute numbers of heat-activated TEs in the corresponding superfamily. The blue dotted line marks the point of overrepresentation (enrichment score >1). **e** The lengths, GC contents, and H3K9me2 levels of TEs. The differences between heat-activated TEs and total TEs in the *Arabidopsis* genome was compared by a two-sided Mann–Whitney *U* test for each feature. For each box plot, center lines indicate the medians; boxes show the 25th and 75th percentiles; whiskers extend to the minimum and maximum. **f** Chromosomal distributions of heat-activated TEs in chromosomes 1 and 2. The uppermost track shows the density of *Copia* (blue) and *Gyspy* (red) retrotransposon superfamilies and DNA transposons (green). The two tracks in the middle show the distribution of retrotransposons and DNA Transposons. Expression differences were calculated by log_10_ (Heat_CPM_ − Control_CPM_). TEs belonging to the *Copia* and *Gyspy* retrotransposon superfamilies and DNA transposons are colored in blue, red, and green, respectively. Other retrotransposons are colored in gray. The bottom track shows the positions of centromeres (black) and pericentromeres (gray).

To genome-wide identify heat-activated TEs, we sequenced the wild-type Col-0 transcriptome under Control, Heat, and Recovery conditions (Supplementary Fig. 1a and “Methods”). Principal component analysis (PCA) using the top 1,000 differentially expressed genes (DEGs) revealed good reproducibility between two biological replicates (Supplementary Fig. 1b). In total, 2,711 genes and 2,056 genes were significantly up- and downregulated, respectively, under Heat (Fig. 1a, Supplementary Fig. 1c, and Supplementary Data 1). Gene ontology (GO) analysis revealed that the upregulated genes showed enrichment for GO terms related to heat. The downregulated genes showed enrichment for GO terms related to temperature stimulus (Supplementary Fig. 1d and Supplementary Data 2). Specifically, many *HEAT SHOCK PROTEIN* (*HSP*) genes (e.g., *HSP70* and *HSP101*) and *small HEAT SHOCK PROTEIN* genes (e.g., *HSP21* and *HSP18.2*) were included in the significantly induced genes (Supplementary Fig. 1c). These results suggest the effectiveness of our heat treatment. Two well-studied epigenetically controlled protein coding genes (PCGs) *SUPPRESSOR OF DRM1 DRM2 CMT3* (*SDC*)^19^ and *QUA-QUINE STARCH*^20^ were induced under Heat (Supplementary Fig. 1c, e), suggesting that the induction of genes by heat stress could be related to epigenetic changes.

It is worth noting that more sequencing reads were assigned to TEs in Heat than that in Control (Supplementary Fig. 2a). Using the same criteria as we used to identify DEGs, we identified 363 upregulated but only 32 downregulated TEs (Fig. 1a and Supplementary Data 3). Thus, unlike the PCGs, which were almost equally upregulated and downregulated, TEs were predominantly activated by Heat (Fig. 1a). Most of the heat-activated TEs (268/363), representing the well-studied *ONSEN*^21^ and *ROMANIAT5*^22,23^ TE families, were completely silenced in Control but showed dramatically high expression in heat (Fig. 1b and Supplementary Fig. 2b). Retrotransposons, such as those in the *Copia* and *Gypsy* superfamilies of long terminal repeat (LTR) retrotransposons and *short interspersed element* non-LTR retrotransposons, were significantly overrepresented in heat-activated TEs (Fig. 1c, d). Conversely, DNA/*MuDR* and RC/*Helitron* transposon superfamilies were underrepresented in heat-activated TEs (Fig. 1c, d). Further TE family enrichment analysis revealed that, in total, 87 of 320 TE families were overrepresented in heat-activated TEs (enrichment score >1 if absolute number ≥3, see Supplementary Fig. 2c). *ATCOPIA78*, which is also known as *ONSEN*^21^ and includes eight full-length *ONSENs* and a partial *ONSEN*, is the most enriched TE family (Supplementary Fig. 2c). Compared with all TEs, heat-activated TEs displayed genetic and epigenetic features including longer sizes, higher GC contents, and higher levels of heterochromatic histone mark H3K9me2 (Fig. 1e), suggesting that heat may preferentially activate heterochromatic transposons. Recently, it was reported that histone H1, the linker histone that binds to the nucleosome entry and exit sites of linker DNA, is required for heterochromatin condensation and perhaps involved in TE silencing^24–26^. Using published H1 chromatin affinity purification sequencing (ChAP-Seq) and MNase-Seq datasets^24,26^ we found that, compared with random selected TEs, heat-activated TEs were enriched by H1.1 and H1.2 and were also occupied by large numbers of nucleosomes (Supplementary Fig. 2d).

Furthermore, a large number (188) of heat-activated TEs were located in pericentromeric and centromeric regions (Fig. 1f and Supplementary Fig. 2e). According to a Fisher’s exact test (among 21,125 TEs on chromosomes arms, 175 are heat-activated. Among 10,064 TEs in pericentromeric and centromeric regions, 188 are heat-activated. The *p*-value is 1.096E−14), the enrichment of heat-activated TEs in pericentromeric and centromeric regions is a true bias (pericentromeric and centromeric regions were defined previously^4^). The heat-activated *Gypsy* superfamily of retrotransposons (155/363) were particularly centered around centromeres. Also, heat-activated retrotransposons in the *Copia* superfamily (23/363) and DNA transposons (41/363) were concentrated in pericentromeric regions, although others were dispersed within euchromatin (Fig. 1f and Supplementary Fig. 2e). Our results were consistent with previous microarray results^14^.

**Fig. 2:**
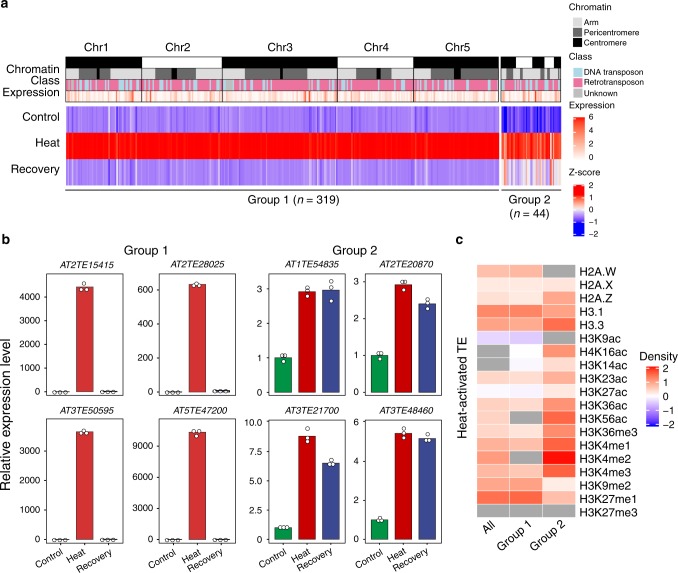
Characterization of two groups of heat-activated TEs. **a** Heatmap of heat-activated TEs in Control, Heat, and Recovery. The chromatin track shows the genomic locations of heat-activated TEs. The class track shows the three classes of the heat-activated TEs. The expression track shows the expression levels of heat-activated TEs in log_2_ (CPM + 1). The bottom three tracks show relative expression levels (*Z*-scores) for Control, Heat, and Recovery and range from −2 (blue) to 2 (red). Most heat-activated TEs, classified into Group 1, are silenced again during recovery. Some heat-activated TEs, classified into Group 2, exhibited persistent activation or were not completely restored during recovery. **b** Expression dynamics of randomly selected Group 1 and Group 2 TEs as determined by RT-qPCR. The expression value of each replicate was normalized to the expression level of Col-0. Circles denote relative expression values. Bars indicate mean values of three technical replicates. **c** The enrichment (red) and depletion (blue) patterns of epigenetic features in Group 1 and Group 2 TEs compared with total TEs in *Arabidopsis*. Relative density ranges from −2 to 2. Gray indicates that the epigenetic feature is neither enriched nor depleted (two-sided Mann–Whitney *U* test, *p* > 0.05). Source data underlying  **b** and **c** are provided as a Source Data file.

Under Recovery, 319 out of 363 heat-activated TEs were resilenced, while the rest remained activated (Fig. 2a). The 319 TEs that were silenced again were referred to as Group 1 TEs and other heat-activated TEs were referred to as Group 2 TEs (Fig. 2a). We validated the expression patterns of representative Group 1 and Group 2 TEs by RT-qPCR (Fig. 2b and Supplementary Fig. 3a, b). We then compared the epigenetic features of Group 1 and Group 2 TEs using publicly available data (see “Methods”) and found that the density of active histone marks, such as H4K16ac, H3K36ac, H3K56ac, H3K36me3, and H3K4 methylation, was higher, but the density of repressive histone marks, such as H3K9me2 and H3K27me1, was lower in Group 2 TEs. This may explain why the activation of Group 2 TEs persisted under Recovery (Fig. 2c and Supplementary Fig. 3c).

### Heat induces rearrangement of chromatin organization

Previous FISH studies revealed that heterochromatin decondensation accompanies the activation of TEs under prolonged heat stress^13^. In our study, H3K27me1 immunostaining results further confirmed that heterochromatin underwent decondensation under Heat and recondensed under Recovery (Supplementary Fig. 4a,b). Furthermore, the nuclei became bigger under Heat (Supplementary Fig. 4c). To test whether chromatin organization rearrangement and TE release under Heat are correlated, we precisely mapped the changes of 3D chromatin organization in response to heat by performing Hi-C experiments using *Arabidopsis* under Control and Heat conditions (Supplementary Data 4 and “Methods”). In total, we detected more than 170 and 195 million interaction pairs under Control and Heat, respectively. The ratios of *cis* (intrachromosomal) to total valid interactions, which are positively correlated with Hi-C quality^27^, reaches 83.4% (Control) and 87.5% (Heat) in our Hi-C datasets, similar to the ratios from in situ Hi-C^28^ and much higher than those from dilution Hi-C^4,5,29–31^ of *Arabidopsis*, thus indicating the high quality of our Hi-C. Genome-wide and chromosome-wide comparisons of eigenvalues, correlation analysis of eigenvalues of the first principal component (PC1) (Supplementary Fig. 5a,b) and direct comparisons of the Hi-C interaction heatmaps of two biological replicates (Supplementary Fig. 5c,d) revealed good reproducibility of our Hi-C. To further assess the reliability of our Hi-C, we compared our Control Hi-C datasets with five previously published Hi-C datasets of wild-type *Arabidopsis thaliana*^4,5,29–31^. Correlation and enrichment analyses of eigenvalues of the PC1 to various genomic and epigenomic features validated the similarity (*r* = 0.99) between our Control dataset and other Hi-C datasets (Supplementary Fig. 6). Finally, by using the pipeline of landmark Hi-C research^32^, the resolution of our Hi-C data was estimated to be 0.95 and 0.85 kb for Control and Heat, respectively (Supplementary Data 4 and “Methods”), which were only reached by a Hi-C map constructed by merging several datasets of *Arabidopsis*^31^.

We then explored genome-wide chromatin interaction patterns of Control and Heat at 100 kb resolution (see “Methods”). Like Hi-C maps constructed for other species and cell types^3^, our Hi-C maps for *Arabidopsis* under Control and Heat conditions show frequent chromatin interactions along the diagonal (Supplementary Fig. 7). These chromatin interaction patterns can be explained by the existence of chromosome territories^27^. Prominent interactions were observed within the pericentromeric regions, among different pericentromeric regions, and among telomeres (Supplementary Fig. 7), which were consistent with previously published Hi-C findings^5^.

**Fig. 3:**
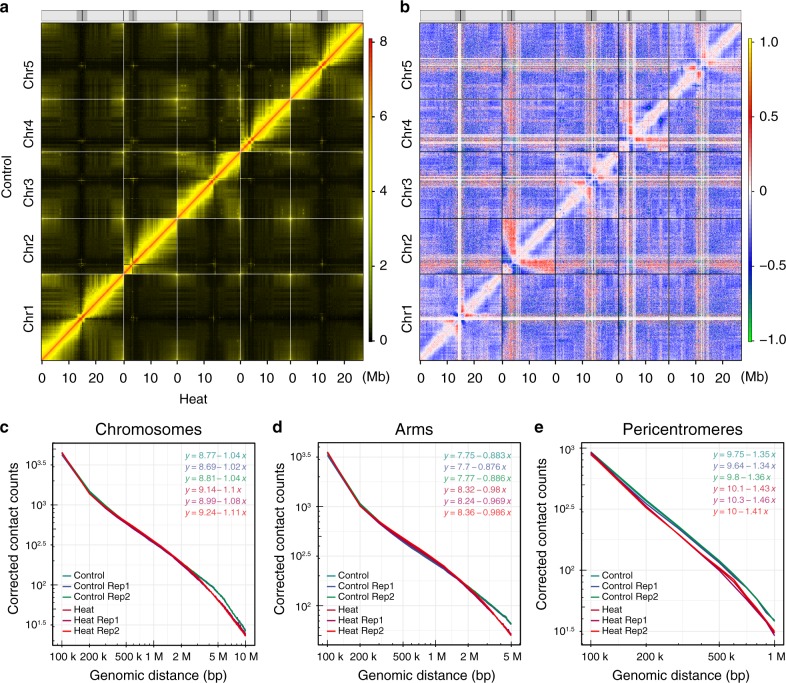
Heat induces rearrangement of chromatin organization. **a** Genome-wide chromatin organization revealed by a Hi-C interaction frequency heatmap at 100 kb resolution for all *Arabidopsis* chromosomes in Control and Heat. Each pixel denotes all interactions between any two 100 kb genomic loci from the linear genome. Intensity represents log_2_ normalized contact frequencies. The top track shows the positions of centromeres (black) and pericentromeres (gray). **b** Genome-wide heatmap of relative interaction differences between Heat and Control. The relative difference is calculated as the difference of two interaction frequencies divided by the mean of two interaction frequencies. In the plot, Hi-C interactions that become stronger (chromosome condensation) in Heat compared with Control are in red to orange, while Hi-C interactions that become weaker (chromosome loosening) in Heat compared with Control are in blue to green. Hi-C interactions that do not change in Heat compared with Control are in white. The genomic bin size is 100 kb and the upper- and lower-triangular matrices are symmetric. The top track shows the positions of centromeres (black) and pericentromeres (gray). **c** Averaged scaling plot of interaction frequencies against increasing genomic distance for all *Arabidopsis* chromosomes. The genomic bin size is 100 kb. **d** Averaged scaling plot of interaction frequencies against increasing genomic distance for all *Arabidopsis* chromosome arms. The genomic bin size is 100 kb. **e** Averaged scaling plot of interaction frequencies against increasing genomic distance for all *Arabidopsis* pericentromeric regions. The genomic bin size is 100 kb.

To investigate the effects of heat stress on global chromatin organization, we calculated relative interaction differences at 100 kb resolution (Fig. 3a, b and Supplementary Fig. 8), where a relative interaction difference was defined as the difference of two interactions divided by the mean of the two interactions^4,29,33^. Visual inspection of the map of relative interaction differences revealed prominently enhanced interactions between pericentromeric regions and distal regions of the chromosome arms in the genome, especially in chromosomes 2 and 4 under Heat, while slightly altered proximal intrachromosomal interactions were found along the chromosomes (Fig. 3b and Supplementary Fig. 8a–e). However, interactions within pericentromeres were greatly reduced. Both distal intrachromosomal interactions and many interactions between different chromosome arms were also reduced (Fig. 3b and Supplementary Fig. 8a–e). These changes can be observed in both biological replicates (Supplementary Fig. 8f). Thus, heat stress could cause global genome organization rearrangement, altering proximal interactions within chromosome arms, as well as interactions of chromosome arms with pericentromeric regions.

Previous research suggested that the average interaction frequency between any two loci decays in a power-law function as the genomic distance increases^4^. To examine this, we generated scaling plots of interaction frequencies against genomic distance for different regions in Control and Heat at 100 kb resolution. Scaling plot results indicated that heat stress caused a decrease of long-range chromatin interactions, which are far away from the diagonal in the Hi-C heatmap (Fig. 3c–e and Supplementary Fig. 9a–c). However, the short-range chromatin interactions, which are near the diagonal, were slightly enhanced at 1 kb resolutions (Supplementary Fig. 10).

To quantitatively assess heat-induced chromatin organization changes, we calculated interaction decay exponents (IDEs), which were defined as the slopes of a linear fit of mean interaction intensities detected at a given range^1^, that represent the distance-dependent decay of interaction frequency at chromosome arms, chromosomes, and pericentromeres (Fig. 3c–e and Supplementary Fig. 9d). IDEs of chromosomes, chromosome arms, and pericentromeres in Control were −1.04, −0.88, and −1.35, respectively, and comparable with previously calculated IDEs in *Arabidopsis*^4^ (Supplementary Fig. 9d). However, IDEs of chromosomes, chromosome arms, and pericentromeres in Heat were −1.10, −0.98, and −1.43, respectively (Supplementary Fig. 9d). In general, heat-stressed chromatin exhibited smaller IDEs in different genomic compartments (Supplementary Fig. 9d).

### Chromatin compartmentation is weakened under heat stress

**Fig. 4:**
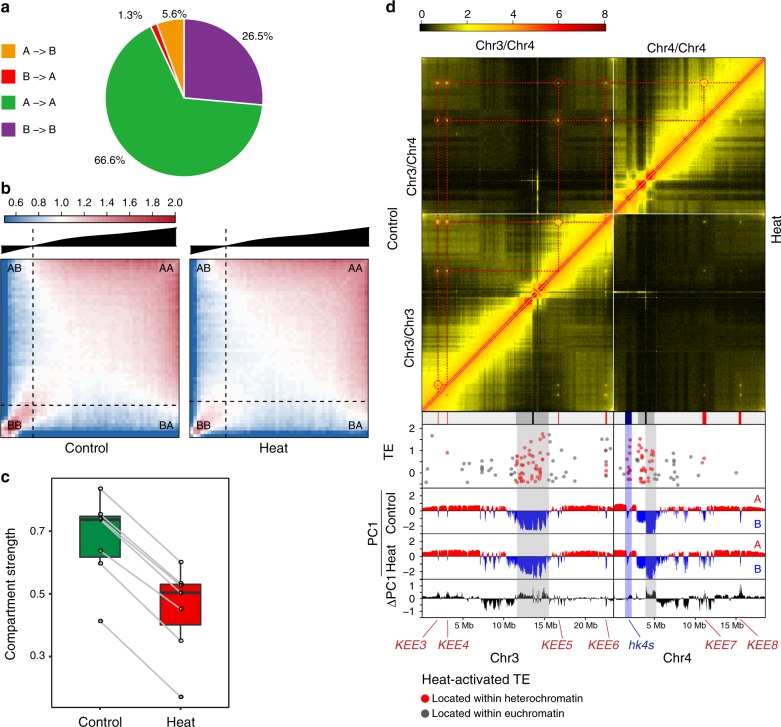
Heat weakens chromatin compartmentation. **a** Pie chart showing the percentages of chromatin compartment switching induced by heat. A compartment: active compartment; B compartment: repressive compartment. **b** Saddle plots of chromatin compartmentalization: mean *cis* observed interaction frequencies divided by expected interaction frequency between 20 kb bins. The plots are ordered by PC1 values from Control. Interactions between A compartments are in the top right, and interactions between B compartments are in the bottom left. **c** Compartment strengths in Control and Heat for each chromosome arm split by centromere. Compartment strength is defined as natural logarithm of interactions of AA + BB normalized by interactions of AB. For the box plot, center lines indicate the medians; boxes show the 25th and 75th percentiles; whiskers extend to the minimum and maximum. **d** Heatmap showing contact frequencies between and within chromosomes 3 and 4 in Control and Heat. The upper- and lower-triangular matrices correspond to Control and Heat, respectively. The values on the diagonal line (interactions between nearby bins) are assigned to zero. The KEEs are highlighted by dashed, red circles and interconnected by dashed lines to indicate the relationships with each other and the positions of KEEs in the linear genome. The track below shows the positions of centromeres (black), pericentromeres (gray), the knob *hk4s* (blue), and KEEs (red). The lower panel shows the positions and differential expression of heat-activated TEs within chromosomes 3 and 4, PC1 values in Control condition, PC1 values in Heat condition, and ∆PC1 values (PC1_Heat_ − PC1_Control_) horizontally. The positive PC1 values indicate A compartments. The negative PC1 values indicate B compartments. The positions of pericentromeres and the knob *hk4s* with increased PC1 values are indicated. Source data underlying **a** and **d** are provided as a Source Data file.

Active (A) and inactive (B) compartments in *Arabidopsis* can be identified by bidirectional PC1 values^4^. Comparison of PC1 values in Control and Heat (Supplementary Fig. 11) revealed that heat only induces minimal A and B compartments switching (Fig. 4a and Supplementary Fig. 11). However, chromatin compartmentation was weakened by heat stress (Fig. 4b, c). The weakening of chromatin compartmentation was prominent in pericentromeric regions and the knob (*hk4s*) (Fig. 4d and Supplementary Fig. 11). The weakening of chromatin compartmentation was associated with an decrease in chromatin interactions within the same compartment (Fig. 4b).

### Interactions between KEEs are reduced under heat stress

**Fig. 5:**
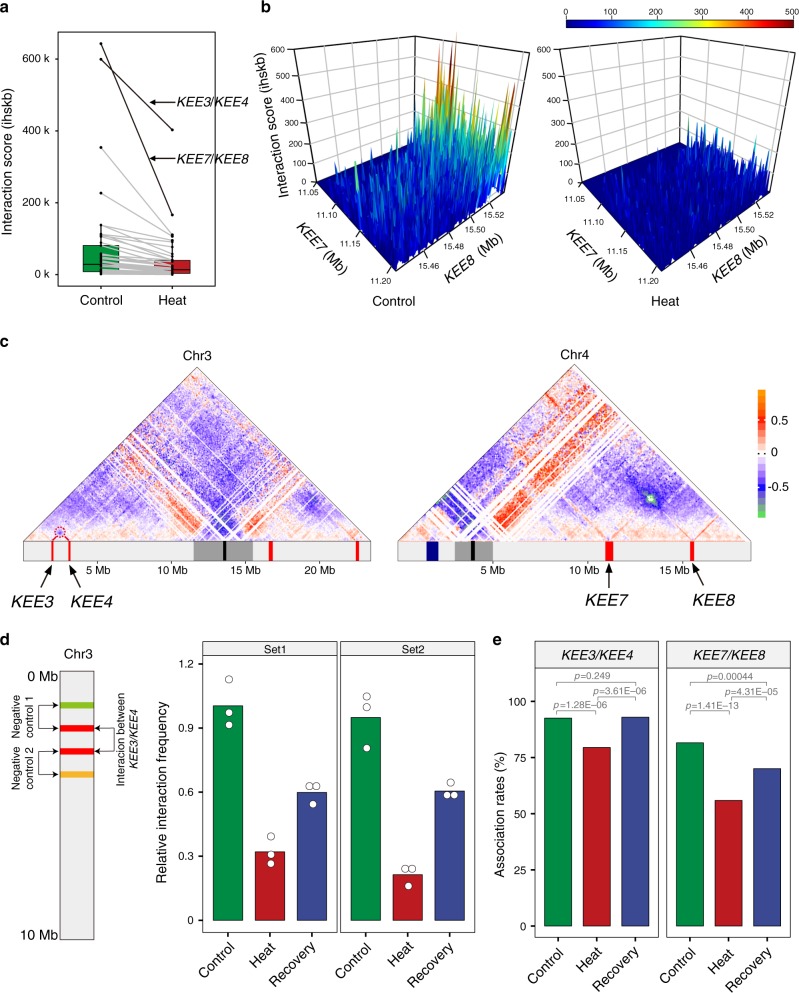
Reduced interactions among KEEs under heat stress. **a** Pairwise interaction scores of all ten KEEs in Control and Heat, defined as the interactions per hundred square kilobases per billion mapped reads (ihskb). *KEE3/KEE4* and *KEE7/KEE8* are highlighted. For each box plot, center lines indicate the medians; boxes show the 25th and 75th percentiles; whiskers extend to the minimum and maximum. **b** Perspective plots of the interactions between *KEE7* and *KEE8* in Control and Heat. The *X*-axis and *Y*-axis show the genomic positions of *KEE7* (Chr4: 11,050,000–11,206,537) and *KEE8* (Chr4: 15,441,465–15,537,500). The *Z*-axis shows the interaction scores. **c** Triangle heatmaps of the relative differences between Heat and Control in chromosomes 3 and 4. Hi-C interactions that become stronger (chromosome condensation) in Heat than those in Control are in red to orange, while Hi-C interactions that become weaker (chromosome loosening) in Heat than those in Control are in blue to green. Hi-C interactions that do not change in Heat compared with Control are in white. The genomic bin size is 100 kb. The interactions of *KEE3*/*KEE4* and *KEE7*/*KEE8* are highlighted with circles. The bottom track shows the positions of centromeres (black), pericentromeres (gray), the knob *hk4s* (blue), and KEEs (red). **d** 3C results showing the significantly decreased interactions between *KEE3* and *KEE4* in Heat compared with that in Control, and the increases in Recovery compared with Heat. Circles denote relative interaction frequency. Bars indicate mean values of three technical replicates. **e** FISH results showing the association rates of *KEE3/KEE4* and of *KEE7/KEE8*. Bars indicate the association rates (“Methods”). Significant differences between two groups at a time were determined by the two-sided Fisher’s exact test, with *p* values adjusted by the false discovery rate method for multiple comparisons. Exact *p* values are shown above the bars. Source data of **d** and **e** are provided as a Source Data file.

Intriguingly, the signals for *KNOTs*, which are formed by chromatin interactions among KEEs, were remarkably reduced (Fig. 4d). We used HOMER^34^ to calculate pairwise interaction scores of the ten KEEs in the *Arabidopsis* genome at 1 kb resolution. Most (29/45) interaction scores between KEEs were reduced by at least 50% (Fig. 5a). The reduction was most prominent for interaction scores between *KEE7* and *KEE8* (Fig. 5a–c), which was highest in Control. As a pair, *KEE3* and *KEE4*, which had the second-highest interaction frequency under Control, also underwent dramatic interaction score reductions under Heat (Fig. 5a, c). To validate changes of interaction frequencies between KEEs, we performed 3C experiments. Our results showed that the relative interaction frequency between *KEE3* and *KEE4* was significantly decreased in Heat compared with that in Control (Fig. 5d). Their interaction was partially recovered in Recovery (Fig. 5d), suggesting that the interaction frequency decrease between *KEE3* and *KEE4* was reversible. We also performed FISH experiments to analyze the association rates of *KEE3*/*KEE4* and *KEE7*/*KEE8* and found those rates to be significantly decreased under Heat and recovered under Recovery (Fig. 5e and Supplementary Fig. 12). Together, our results suggest that the *KNOT* is a dynamic structure.

### Heat stress causes extensive knob related chromatin decondensation

Zooming in on the first 6 megabases of chromosome 4, we also found two distinctly reduced chromatin interactions in the heatmap. First, chromatin interactions within the knob (Chr4:1,560,561-2,082,885) in chromosome 4 showed a distinct decrease, which can be seen visually (Supplementary Fig. 13). Then, a virtual 4C profile generated from the Hi-C contact matrix was plotted from a site (Chr4:1,760,000-1,780,000) within the knob that contains a previously reported viewpoint (Chr4:1,765,891) from 4C^11^ (Supplementary Fig. 13). A virtual 4C profile, as well as a heatmap, demonstrated that a mid-range chromosome loop, possibly mediated by long-range chromatin interactions between the knob and the right-half of the pericentromere (Chr4:3,856,518-5,000,000), had remarkably reduced interaction intensities in Heat (Supplementary Fig. 13). Our results indicated that, like the *KNOT* interactions, the interactions within the knob and interactions between the knob and the right-half of the pericentromere were also reduced under Heat.

### Heat activation of TEs rises as chromatin interactions drop

To evaluate the relationship between the activation of TEs and chromatin organization changes in response to heat stress, we compared changes of TE expression levels with changes in chromatin compartmentalization. The activation of TEs often coincided with an increase in PC1 value in Heat condition relative to Control condition in heterochromatin (Fig. 4d and Supplementary Fig. 11). Specifically, among 156 heat-activated TEs in euchromatin, only 51 (32.7%) were associated with an increase in PC1 value. However, among 209 heat-activated TEs in heterochromatin, 135 (64.6%) were associated with an increase in PC1 value. For heat-activated TEs in pericentromeric regions and the knob, the percentage was even higher. We further compared changes of TE expression levels with changes in knob-mediated interactions. Heat-induced TE activation correlated with reduced chromatin interactions within the knob and the mid-range chromosome loop between the knob and the left pericentromere of chromosome 4 under heat stress. Eleven heat-activated TEs resided in regions that form the knob (Supplementary Fig. 13 and Supplementary Data 3).

### Local chromatin interaction changes associate with TE activation

PCA analysis used 20 kb bin, which is much longer than lengths of TEs, including heat-activated TEs (Fig. 1e). Comparison between local chromatin organization changes and heat-induced TE activation is necessary to better determine the level of correlation between chromatin reorganization and heat-induced TE activation. To this end, we first analyzed the relationship between heat-activated TEs and small chromatin loops (2–25 kb scale) identified from a previous study^31^. These small chromatin loops were identified from *Arabidopsis* genome except regions near centromeres (Chr1:13,698,787-15,897,560; Chr2:2,450,002-5,500,000; Chr3:11,298,762-14,289,014; Chr4:1,800,001-5,150,000; Chr5:10,999,995-13,332,770). We determined whether heat-activated TEs overlap with loops, that is whether a TE body overlaps with one end (anchor) of a chromatin loop or a TE body is inside a chromatin loop. We found that 131 heat-activated TEs overlapped with at least one chromatin loop (Supplementary Data 5). The results suggest the existence of local chromatin structures over most heat-activated TEs. Interestingly, when we order heat-activated TEs by the numbers of their overlapped chromatin loops, we found that many TEs overlapped with dozens of chromatin loops (Supplementary Data 5). For example, *AT3TE13060* from *KEE4* and *AT5TE17345* from *KEE9* overlapped with ~30 chromatin loops.

**Fig. 6:**
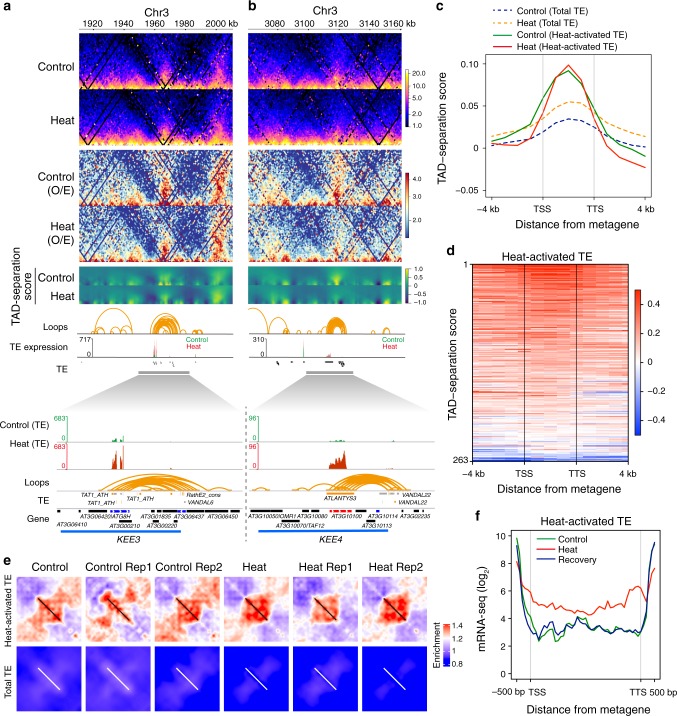
Chromatin organization rearrangement revealed by high-resolution Hi-C correlates with heat-induced TE expression. **a**, **b** Changes of local chromatin organization and TE expression at *KEE3* and *KEE4* loci after heat treatment. Two 100 kb regions (depth: 50 kb) containing *KEE3* and *KEE4*, respectively, are shown. The top panels are heatmaps showing natural logarithm of 1 kb Knight and Ruiz (KR) normalized contact frequency in Control and Heat. Below are heatmaps (O/E) showing natural logarithm of observed/expected contact frequency in Control and Heat at 1 kb resolution. Below are heatmaps showing TAD-separation scores at different window sizes for each Hi-C genomic bin in Control and Heat. Loops were collected and transformed from a previous study (“Methods”). TE expression track shows the expression levels of TEs in Control and Heat. Their expression levels in Control and Heat are under the same scale and overlayered together. Only TE expression is shown. The expression of PCGs is not shown (“Methods”). The expanded panels show TE expression and the corresponding chromatin loops over *KEE3* and *KEE4*. **c** Metagene plot of TAD-separation scores over bodies of heat-activated TEs and total TEs and flanking regions. Only TEs with length over 1,000 bp are considered. TSS transcription start site, TTS transcription termination site. **d** Heatmap of TAD-separation scores over bodies of 263 heat-activated TEs and flanking regions in Control. **e** Local rescaled pileup of chromatin organization over bodies of heat-activated TEs (upper panel) and total TEs (lower panel) in Control, Heat, and different biological replicates. The values are normalized by a local background (“Methods”). The diagonal bars are the locations of scaled heat-activated TEs (upper panel) and total TEs (lower panel). **f** Metagene plot of RNA-Seq read coverage over bodies of heat-activated TEs in Control, Heat, and Recovery. Heat stress can activate heterochromatin-associated transposon elements (TEs). Here, the authors show that heat stress leads to global rearrangement of 3D genome and TEs activation closely correlates with 3D chromatin organization rearrangement in *Arabidopsis*.

Secondly, to show fine-scale chromatin organization changes upon heat, we generated Hi-C chromatin interaction map (in Knight and Ruiz (KR) balanced matrix and observed/expect matrix which eliminates distance effects) at 1 kb resolution. We found prominent chromatin interaction changes at specific loci like *KEE3*, *KEE4*, *KEE6*, and *KEE9* (Fig. 6a, b and Supplementary Fig. 14a–c). We examined TE expression changes at these loci. We found that TE activation was correlated with chromatin organization changes (Fig. 6a, b and Supplementary Fig. 14c). It should be noted that these loci contain heat-activated TEs overlapping with large numbers of chromatin loops (Fig. 6a, b and Supplementary Fig. 14).

Thirdly, we found that small triangles are widespread and prominent in *Arabidopsis* Hi-C map at kb level resolution (Fig. 6a, b and Supplementary Fig. 14), indicating the prevalence of local 3D chromatin structures in *Arabidopsis*. These small triangles overlapped with previously identified chromatin loops^31^. Those triangles/chromatin loops were not topologically associating domains (TADs). They were more like “crumples” defined in yeast by Micro-C^35^. However, because they were similar to TADs in shape, we applied the TAD-separation score method to analyze *Arabidopsis* Hi-C map. The TAD-separation score method has been used to define TADs. Regions with local higher TAD-separation scores are considered to be TAD bodies^36^. We could observe clear triangle structures associated with local peaks of TAD-separation score (Fig. 6a, b and Supplementary Fig. 14). A meta-TE analysis of TAD-separation scores over heat-activated TEs also suggested the existence of local triangle structures over heat-activated TEs (Fig. 6c, d).

Fourthly, to further study the relationships between TE expression and chromatin organization change at a genome-wide scale, we analyzed the chromatin interaction changes associated with TE bodies and flanking regions. We created two-dimensional local rescaled pileup of chromatin interaction frequency relative to local background on heat-activated TEs or flanking regions in Control and Heat. We found that chromatin interactions in upstream and downstream flanking regions of heat-activated TEs were reduced, while chromatin interactions over TE bodies remained unaltered or became stronger (Fig. 6e). Heat-induced TE activation correlated with these chromatin organization changes (Fig. 6e, f). The results from both replicates reached the same conclusion (Fig. 6e).

In summary, chromatin interactions around TE bodies were weakened by heat stress. Heat-induced TE activation correlated with chromatin organization changes (Fig. 6e, f).

## Discussion

In this study, we profiled the changes of gene and TE expression levels in response to heat and then during recovery from heat. We found that many heterochromatic TEs were activated and most of the TE activation events were transient. By performing Hi-C, 3C, and FISH, we demonstrated that heat-induced chromatin organization rearrangement with decreased long-range chromatin interactions but increased local chromatin interactions. We established that TEs were activated where chromatin organization was rearranged.

Since heat-induced TE activation is accompanied by heterochromatin decondensation, we aimed to find the correlation between heat-induced heterochromatin decondensation and heat-induced TE activation at a genome-wide scale. Our Hi-C results revealed that heat could induce genome-wide increases of short-range chromatin interactions but decrease long-range chromatin interactions, and the interactions mediated by two prominent chromatin structures, the *KNOT* and the knob, were also decreased under heat stress. Importantly, we found that decreases of chromatin interactions within pericentromeric regions, the *KNOT*, the knob, and between knob and pericentromeric regions, and within the upstream and downstream flanking regions of heat-activated TEs correlated well with TE activation by heat. In mouse, the release of the murine endogenous retroviral element (MuERV-L/MERVL) family of TEs drives the establishment of insulating TAD boundaries during early embryogenesis^37^, suggesting that heterochromatin decondensation could be a consequence of TE activation. However, the upregulation of TEs other than MuERV-L/MERVL are not coupled with changes in chromatin organization^37^. Furthermore, the activation of TEs in the *Arabidopsis mom1* mutant are not associated with changes in chromatin organization^5^. Thus, chromatin organization rearrangement does not always follow TE activation and it is possible that heterochromatin decondensation gives rise to TE activation.

In an interspecific hybrid of *A. thaliana* and its closest relative *A. lyrata*, the interactions among KEEs became weaker^28^. Here we found another scenario in which the interactions among KEEs are reduced. We found that the interactions within the knob and between the knob and the left pericentromere of chromosome 4 were reduced. We also found that triangle structures, which overlap with small chromatin loops, are prevalent in the *Arabidopsis*. So far, the mechanisms underlying the formation of the *KNOT*, the knob, small chromatin loops, and triangle structures are unclear. In mammals, distinct TADs and loops are formed by cohesin through loop extrusion until CCCTC-binding factor (CTCF) is encountered^38^. *Arabidopsis* REC8 (a core component of cohesin) is enriched in both centromeres and heterochromatin during meiosis^39^. When REC8 is depleted, many transposons are activated^39^. While CTCF has no direct homolog in plants, rice Hi-C analysis revealed that the plant-specific proteins TCP and bZIP recognize motifs embedded in TADs and contribute to the formation of TADs^3,40^. It will be interesting to determine whether REC8, TCP, and bZIP are involved in the formation of the *KNOT* and the knob.

We found that heat stress induces an increase in nuclear size (Supplementary Fig. 4c). However, heat not only induces weakening of some chromosome interactions, but also induces strengthening of some other chromatin interactions and HIT4 is required for heat-induced TE activation^16,17^, suggesting that heat-induced chromatin reorganization and TE activation are active processes. They are not merely a result of bigger nuclei.

Histone H1 binds to linker DNA wrapped away from the nucleosome core particle to form the chromatosome^41^. The establishment of higher-order chromatin structures requires histone H1^41^. Disassociation of H1.2 from chromatin leads to global chromatin decondensation during mouse cell reprogramming to pluripotency^42^. Histone H1 can regulate transposon activity in *Drosophila* ovaries^43^ and in cancer cells^44^. In *Arabidopsis*, histone H1 is required for chromatin condensation, but the function of histone H1 in TE silencing is still unclear^24–26^. Here, we found that heat-activated TEs showed enrichment of H1.1 and H1.2 under normal conditions (Supplementary Fig. 2d). Interestingly, *H1.1* and *H1.2* were downregulated by heat, as revealed by our RNA-Seq and RT-qPCR data (Supplementary Fig. 1c, e). We speculate that the downregulation of histone H1 may contribute to heat-induced chromatin reorganization and TE activation.

In summary, we examined heat-induced TE activation and 3D genome reorganization at high resolution and found a strong correlation between TE activation and chromatin reorganization. Further studies are required to determine whether a cause-and-effect relationship can be established between these two events and dissect the molecular mechanisms upstream and downstream of these two events.

## Methods

### Plant material and growth conditions

Wide-type Col-0 was used for all of the experiments. Plants were grown on 1/2 Murashige and Skoog plates with 1% sucrose and 0.7% agar. For the heat treatment, 7-day-old seedlings were placed in an incubator set at alternating temperatures of 37 and 22 °C, 12 h each, for 3 days with a 12 h photoperiod. For recovery, seedlings that underwent heat treatment were grown under normal growth conditions (a constant 22 °C with a 12 h photoperiod) for an additional 3 days (Supplementary Fig. 1a).

### Analysis of TE expression by RT-qPCR

We extracted total RNA from seedlings using TRIzol reagent (Invitrogen) and removed DNA contamination by using DNase (Ambion). Pure RNA (2 μg) was used for first-strand cDNA synthesis with the PrimeScript II first-strand cDNA synthesis kit (Takara). The resultant cDNA reaction mixture was then diluted five times, and 1 µL was used as the template in a 25 μL PCR reaction with iQ SYBR Green Supermix. *ACTIN7* was used as an internal control and our primers are listed in Supplementary Data 6.

### Immunofluorescence

We performed immunofluorescence as previously described^45^. Briefly, seedlings (2 g) were cut into pieces and homogenized in the cold (4 °C) with 2 mL nucleic extraction buffer (10 mM Tris-HCl (pH 9.5), 10 mM KCl, 250 mM sucrose, 3.97 mM spermidine, 9.86 mM spermine, 0.1% β-mercaptoethanol, and 1% Triton X-100). To fix the nuclei, 2 mL of 8% formaldehyde was added to the homogenate and the sample was incubated at 4 °C for 1 h with constant shaking. The sample was filtered sequentially through 100, 53, and 20 μm filters and then centrifuged at 4 °C. The pellet was resuspended in 300 μL of nucleic extraction buffer with 4.27% sucrose and then added on top of 300 μL of nucleic extraction buffer with 29% sucrose. After centrifugation at 4 °C for 30 min, the white pellet was resuspended in 40 μL of nucleic extraction buffer. Nuclei were placed on histological slides and then immunostained with anti-H3K27me1 (07-448; Millipore; 1:200 dilution) and then incubated with rabbit Alexa488- (A23220; Abbkine; 1:200 dilution) conjugated secondary antibodies for 2 h at 37 °C. After PBS washes, DNA was counterstained using DAPI in Prolong Gold (Invitrogen). Nuclei were observed with a Nikon A1RSi+ confocal microscope. For each sample, about 300 nuclei were analyzed.

### Chromatin conformation capture

Chromatin conformation capture (3C) assays were performed according to previously described procedures^46^. Briefly, seedlings (1–3 g) were cross-linked with 1% formaldehyde in NIB buffer [20 mM HEPES, pH 8.0, 250 mM sucrose, 1 mM MgCl_2_, 5 mM KCl, 40% (v/v) glycerol, 0.25% (v/v) Triton X-100, 0.1 mM phenylmethanesulfonyl fluoride (PMSF), 0.1 mM DTT] and 2 M glycine was used to quench formaldehyde. Seedlings were then washed with ddH_2_O. After removing water and being dried in air, different seedlings in equal weight were ground into fine powder in liquid nitrogen. Nuclei were isolated and then treated with SDS. Chromatin was digested by *Hind*III and DNA ends of digested chromatin were then ligated by T4 DNA ligase. DNA was purified after reverse cross-linking. Then 3C quantitative PCR was performed. The sequences of primers were as previously reported^5^ and listed in Supplementary Data 6. A couple of primers with opposite directions which could amplify a length of 100 bp sequence across one of the *Hind*III restrict site of *SDC* were used as a normalization control.

### Fluorescence in situ hybridization (FISH)

We performed FISH as previously described^47^. Briefly, four bacterial artificial chromosome (BAC) clones were used as probes to monitor chromosome pairing rates in Control, Heat, and Recovery, respectively. F24P17, T22K18, F10M6, and F21C20 represent *KEE3*, *KEE4*, *KEE7*, and *KEE8*, respectively. The BAC DNA was labeled with either biotin or digoxigenin and biotin-labeled probes were detected with Alexa Fluor 488 streptavidin while digoxigenin-labeled probes were detected with rhodamine anti-digoxigenin. We counterstained nuclei with DAPI and acquired chromosome images with a Zeiss A2 fluorescence microscope with a micro-charge-coupled device camera. Paring rates of two BAC clones were defined as 2 (pairing), 1 (half-pairing), and 0 (no pairing). The association rate was calculated as the sum of all the pairing events divided by twofold of the total nuclei number. Distance between two closest probes was measured by Zeiss ZEN lite.

For a post-hoc analysis, we analyzed *KEE* association data using pairwise Fisher’s exact tests with *p* values adjusted by the false discovery rate method for multiple comparisons^48^.

### RNA-Seq and data analysis

We extracted total RNA from seedlings by using the RNeasy Plant Mini Kit (Qiagen) with RNase-Free DNase (Qiagen). Poly-A RNA-Seq library preparation and high-throughput sequencing were performed by Beijing Novogene Co. Ltd and we used the NEBNext® Ultra™ RNA Library Prep Kit for Illumina® (NEB, USA) to generate sequencing libraries, following the manufacturer’s recommendations. The libraries were sequenced by the Illumina Hiseq 4000 platform, generating paired-end, 150 bp reads.

Adapter sequences and poor-quality reads were removed by trim_galore (https://www.bioinformatics.babraham.ac.uk/projects/trim_galore/) with flags —paired and —length 70 (minimum read length after trimming). Then the clean reads were mapped to the *Arabidopsis* reference genome (TAIR10, https://www.arabidopsis.org) guided by Araport11 annotations^49^ (https://www.araport.org) and using TopHat2^50^ with default parameters, except that options —min-intron-length and —max-intron-length were set to 20 and 5000, respectively. Reads were then sorted, indexed and compressed by SAMtools^51^ and only uniquely mapped reads were kept for the following analysis by SAMtools^51^. Here multi-mapped reads were disregarded because it is difficult to determine their real locations, whereas uniquely mapped reads provide sufficient coverage and mappability. We generated Bigwig files by using bam2wig.py with options —u (skip non-unique hit reads) and —wigsum = 10,000,000,000 from RseQC^52^.

TEs with more than 20% of their regions overlapping with genes were removed because of the difficulty of their reads assignment. First, we used featureCounts^53^, with the option —p (fragments will be counted rather than reads), to count over meta-features, like genes or TEs, and then used DESeq2^54^ for differential expression analysis of genes and TEs. The expression levels of genes and TEs in different groups (Heat vs. Control and Recovery vs. Heat) were compared with strict criteria (*q* < 0.05 and fold change >2) and reproducibility was revealed with PCA, using the expression levels of the top 1,000 DEGs. Expression heatmaps were generated by using ComplexHeatmap^55^ in R. Also, in R, we used gtrellis^58^ to illustrate the chromosome-wide distribution of released TEs. The TE family and superfamily information was downloaded from TAIR10 (https://www.arabidopsis.org) and screenshots of typical loci were generated using the Integrative Genomics Viewer (IGV)^56^. GO analyses were conducted by agriGO V2.0 (http://systemsbiology.cau.edu.cn/agriGOv2/index.php) with default parameters.

### Hi-C library construction and sequencing

Annoroad Gene Technology Co., Ltd (Beijing, China) constructed and sequenced our Hi-C libraries as follows. Briefly, the aerial parts of 10-day-old seedlings were collected, cut into pieces, and cross-linked in 2% formaldehyde solution for 15 min and then glycine was added to quench the cross-linking reaction. After the nuclei were isolated, the chromatin was digested^57^. To isolate the interacting chromatin fragments, the cross-linked DNA fragments were end-labeled with biotin-14-dCTP and end joined^58^. Proteins were removed with proteinase K and the DNA was purified using phenol and chloroform. Biotin-14-dCTP was removed from non-ligated fragments with T4 DNA polymerase, then the ligated fragments were sonicated into fragments of 200–600 bp each and the DNA fragments with biotin labeling were enriched using streptavidin C1 magnetic beads. A-tails were added by Klenow (exo-) and Illumina paired-end sequencing adapters were ligated to DNA fragments using a ligation mix. After 12–14 cycles of PCR, the Hi-C libraries were sequenced by an Illumina HiSeq X Ten instrument with 2 × 150 bp reads.

### Hi-C sequencing data processing by HiC-Pro

Fastp^59^ was used to filter low-quality reads and trim adapters from raw Hi-C sequencing reads, then Hi-C sequencing data mapping, reads pairs filtering, and identification of valid ligations were performed in HiC-Pro^60^. To begin, each clean mate from the read pairs was independently mapped to the *Arabidopsis* reference genome, TAIR10 (https://www.arabidopsis.org), by bowtie2^61^ using an end-to-end method to avoid linear constraints between read pairs. A two-step approach in HiC-Pro was used to rescue a small number of chimeric reads which had at least one read spanning over the junction of the ligation product. Singletons, low-quality mapped reads, and multiple mapped reads were discarded, and only unique mapped reads were kept for downstream analysis. Based on the sequence of reference genome and the restriction enzyme, each mapped read was assigned to a restriction fragment. By excluding both dangling-end and self-circle pairs, invalid ligation products were separated from valid pairs. We used the valid read pairs that comprised two different restriction fragments to construct our downstream Hi-C contact map. After removing read pairs from PCR duplicates, we calculated Hi-C resolution based on previous definition^32^ and then constructed Hi-C contact maps with equal-sized bins for different resolutions. HiC-Pro contact matrixes and bins were transformed into different formats for specific analysis. Hi-C contact matrixes at different resolutions were imported into HiCExplorer^62^ (https://github.com/deeptools/HiCExplorer/) in a format h5 by hicTransform with fixed bins. Cooler files were generated by hicTransform.

### Hi-C map normalization and comparisons

We normalized Hi-C contact maps in HiCdat^33^, correcting our Hi-C data biases with the implicit bias-removal method: iterative coverage normalization^63^. To assess differences between Hi-C replicates, in-house generated Hi-C datasets, and previously published Hi-C datasets, multiple functions in HiCDat^33^ were used, including f.HiC.correlation.matrix, and f.principle.component.analysis.and.features. To compare two Hi-C heatmaps, we calculated relative differences as the difference of matched bins between the two samples, further divided by the mean contact interaction frequencies between the two samples^29^. Comparisons between Control, Control Rep1, Control Rep2, Heat, Heat Rep1, and Heat Rep2 were performed.

To quantitatively detect the difference in chromatin organization between samples, distance-dependent decay of interaction frequency was calculated by a modified version of hicPlotDistVsCounts from HiCExplorer^62^. The decay plots were visualized by ggplot2 (http://ggplot2.tidyverse.org). The IDE was defined as the slope of a linear fit to the decreased mean interaction frequency with increased genomic distance and it was used to quantify the differences of chromatin organizations among different genomic compartments or different samples^4^. To further examine the effects of heat on chromatin organization, linear regression on averaged raw decay curves was performed to calculate IDEs. To compare changes in *KEE*-mediated chromatin interactions at 1 kb resolution, we used HOMER^30^ to calculate interaction scores (interactions per hundred square kilobases per billion mapped reads) among pairwise interactions of KEEs.

To define and compare chromatin compartments in Control and Heat, PCA analyses of Hi-C were performed by HOMER (http://homer.ucsd.edu/homer/interactions2/index.html). To begin, The PCA calculation was performed on individual chromosome arms separated by centromeres. A balanced and distance normalized matrix was created using window size of 60 kb binned every 20 kb for each individual chromosome arm, reporting observed/expected matrix. Then, a correlation matrix was generated by calculating correlation coefficient. The matrix was further analyzed by the prcomp function from R, and the eigenvalues for each 20 kb region along the PC1 were assigned to each region (PC1 values). To make PC1 values more comparable across chromosome arms, the PC1 values of each chromosome arm were also scaled by their standard deviation. For each chromosome, the orientation of PC1 values is determined by H3K4me3 ChIP-Seq peaks to ensure positive PC1 values associated with A compartments and negative PC1 values with B compartments. Differences in PC1 values (∆PC1) in Heat relative to Control conditions were calculated by subtractBedGraphs.pl in HOMER with option —center. Chromatin compartmentation saddle plot and chromatin strength were calculated by GENOVA (https://github.com/robinweide/GENOVA/) using 20 kb contact matrix.

### Comparison between Hi-C data and TE expression data

All Hi-C datasets were trimmed to the same sequencing depth by hicNormalize from HiCExplorer^62^. The raw matrix was further transformed into normalized Hi-C contact matrix by KR balancing method by hicCorrectMatrix from HiCExplorer^62^. Hi-C observed/expected matrixes were then generated by hicTransform using a method of obs_exp_lieberman. Figure 6a, b and Supplementary Fig. 14 were generated by pyGenomeTracks (https://github.com/deeptools/pyGenomeTracks/) based on Hi-C data, RNA-Seq data, and annotations. Configure files were submitted into github (https://github.com/Linhua-Sun/Ath_Heat_Hi-C). Enlarged expression tracks were generated by IGV. TE expression tracks were extracted by bedops^64^.

TAD-separation score is used to define the degree of segregation between left and right part of each Hi-C genomic bin. In our study, we found that local triangles are widespread and prominent in *Arabidopsis* Hi-C map, indicating the prevalence of local 3D chromosome structures in *Arabidopsis*. To test whether heat-activated TEs are associated with local chromosome structures, we applied hicFindTADs from HiCExplorer to calculate TAD-separation scores with options —minDepth 3000 —maxDepth 30000 —step 1000 —thresholdComparisons 0.05 --delta 0.01 --correctForMultipleTesting fdr at 1 kb resolution in Control and Heat. Metagene plot and associated heatmap of TAD-separation scores were generated by seqplots (http://seqplots.ga/). And then local rescaled pileup plots normalized by local backgrounds were generated by coolpup.py^65^ (https://github.com/Phlya/coolpuppy/) with options —minshift 10000 —maxshift 50000 —rescale —local —rescale_size 87.

### Public genomic data analysis

Various *Arabidopsis* ChIP-seq and DNase-seq datasets were downloaded from the Plant Chromatin State Database^66^ and the average occupied score for each element was calculated by bedmap^64^ with option —wmean. ChIP-seq occupied scores among different groups were then evaluated by pairwise Mann–Whitney *U*-tests with Bonferroni–Holm method for multiple comparisons^67^. Used ChIP-Seq, MNase-Seq, and ChAP-Seq datasets were summarized in Supplementary Data 7.

Chromatin loops were collected and transformed from previously published study^31^. The significant chromatin loops were called from regions except near centromeres (Chr1:13,698,787-15,897,560; Chr2:2,450,002-5,500,000; Chr3:11,298,762-14,289,014; Chr4:1,800,001-5,150,000; Chr5:10,999,995-13,332,770). To compare these small chromatin loops and heat-activated TEs, we used bedtools pairToBed with option —type ospan.

### Reporting summary

Further information on research design is available in the Nature Research Reporting Summary linked to this article.

## Supplementary information

Supplementary Information

Reporting Summary

Description of Additional Supplementary Files

Supplementary Data 1

Supplementary Data 2

Supplementary Data 3

Supplementary Data 4

Supplementary Data 5

Supplementary Data 6

Supplementary Data 7

## Data Availability

Data supporting the findings of this work are available within the paper and its Supplementary Information file. A reporting summary for this Article is available as a Supplementary Information file. The datasets generated and analyzed during the current study are available from the corresponding author upon request. Raw Hi-C sequencing reads that support the findings of this study have been submitted to the NCBI Sequence Read Archive under accession number PRJNA545383. RNA-Seq sequencing reads have been submitted to Gene Expression Omnibus under accession number GSE132415. The source data underlying Figs. 2b, c, 4a, d, 5d, e, and Supplementary Figs. 1d, 3a, b, 4b, c, 9d, and 12b are provided as a Source Data file.

## Source Data

Source Data

## Code availability

All related scripts have been uploaded into github.
