# Supplementary material for: Heat stress-induced transposon activation correlates with 3D chromatin organization rearrangement in Arabidopsis
Source: Nat Commun. 2020 Apr 20;11:1886. doi: 10.1038/s41467-020-15809-5 (PMC7170881; doi:10.1038/s41467-020-15809-5)
Supplement: Supplementary file 1 — Supplementary Information [file 41467_2020_15809_MOESM1_ESM.pdf]

Heat stress-induced transposon activation correlates with 3D chromatin organization rearrangement in *Arabidopsis*

Sun *et al.*

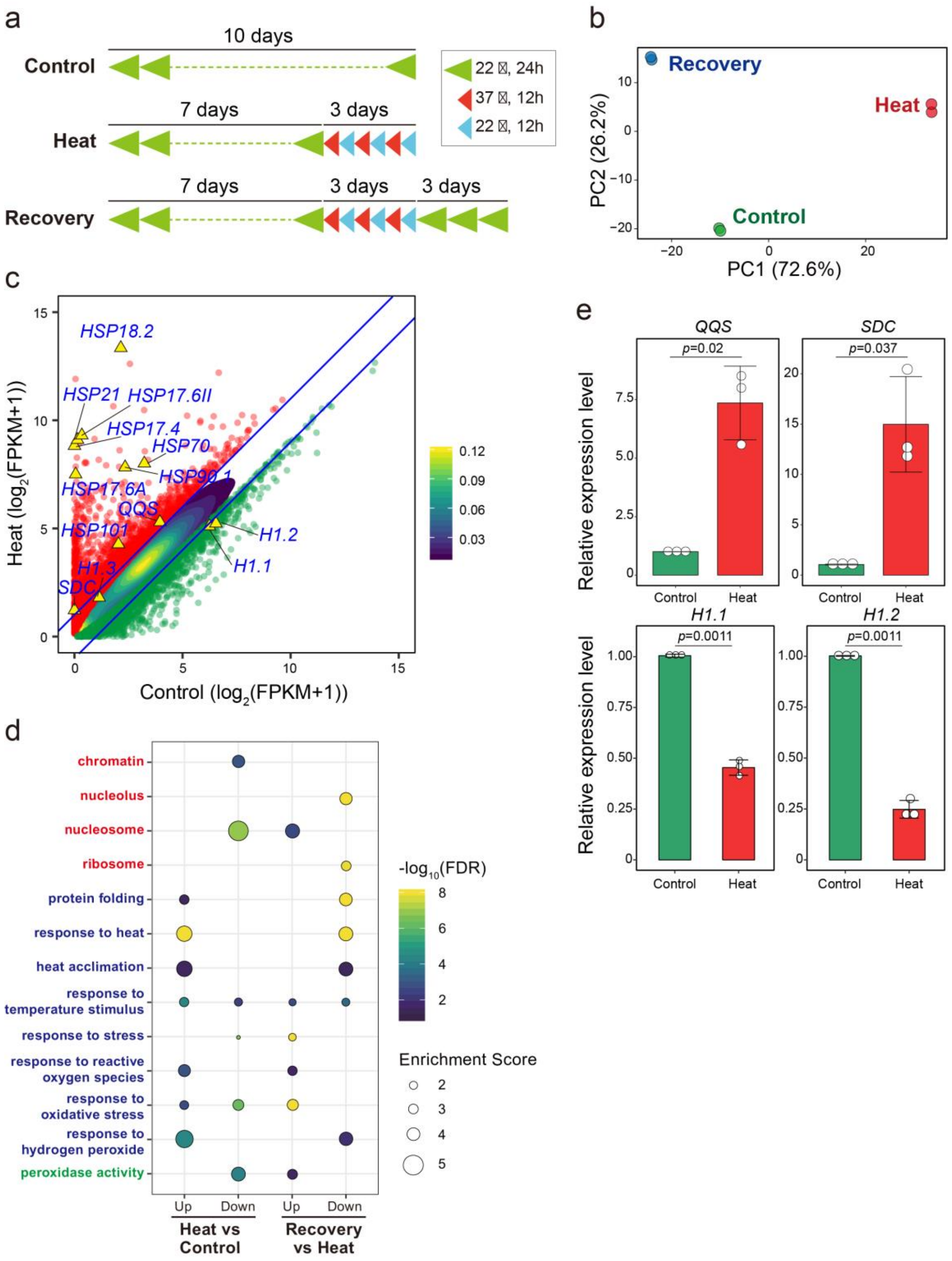

Supplementary Figure 1. RNA-Seq analysis of seedlings subjected to different treatments.

a. Experimental conditions of the Control, Heat, and Recovery treatments of *Arabidopsis thaliana* seedlings. Control: 22 °C for 10 days; Heat: 22 °C for 7 days, then 37 °C/22 °C for 3 days; Recovery: 22 °C for 3 days after Heat. All groups were cultured under long photoperiod conditions.

b. Principal components analysis of the 6 samples using the top 1,000 differentially expressed genes. The X-axis shows the first PC and its variance proportion and the Y-axis shows the second PC and its variance proportion.

c. Scatter plot of differentially expressed genes in Heat vs Control from RNA-seq data. FPKM: Fragments Per Kilobase Million. Red points: up-regulated genes after heat stress. Green points: down-regulated genes after heat stress. Yellow triangle: typical genes altered after heat stress. The blue lines indicate two-fold change. The underling 2D kernel density estimation plot shows the density of unchanged genes.

d. Gene Ontology (GO) analysis of the significantly up-regulated and down-regulated genes in Heat vs Control and in Recovery vs Heat. X-axis shows different comparisons, and Y-axis shows the selected GO terms. Red GO terms denote cellular component. Blue GO terms denote biological process. Green GO terms denote molecular function. The color of the circles represents the value of $-\log_{10}$ (FDR). The size of the circles represents enrichment score of the corresponding GO term. Enrichment score is calculated as (NO of input genes in a GO terms / NO of input genes) / (NO of genes in a GO terms/ NO of total genes).

e. RT-qPCR verified the expression level of *QQS*, *SDC*, *H1.1*, and *H1.2*. The expression value of each replicate was normalized to the expression level of Col-0. Circles denote relative expression values. Data are presented as mean values +/- standard deviation (SD) from three biological replicates. (Two-sided Student's t-test, exact *p-values* are added on the bars). Source data of Supplementary Figure 1d are provided as a Source Data file.

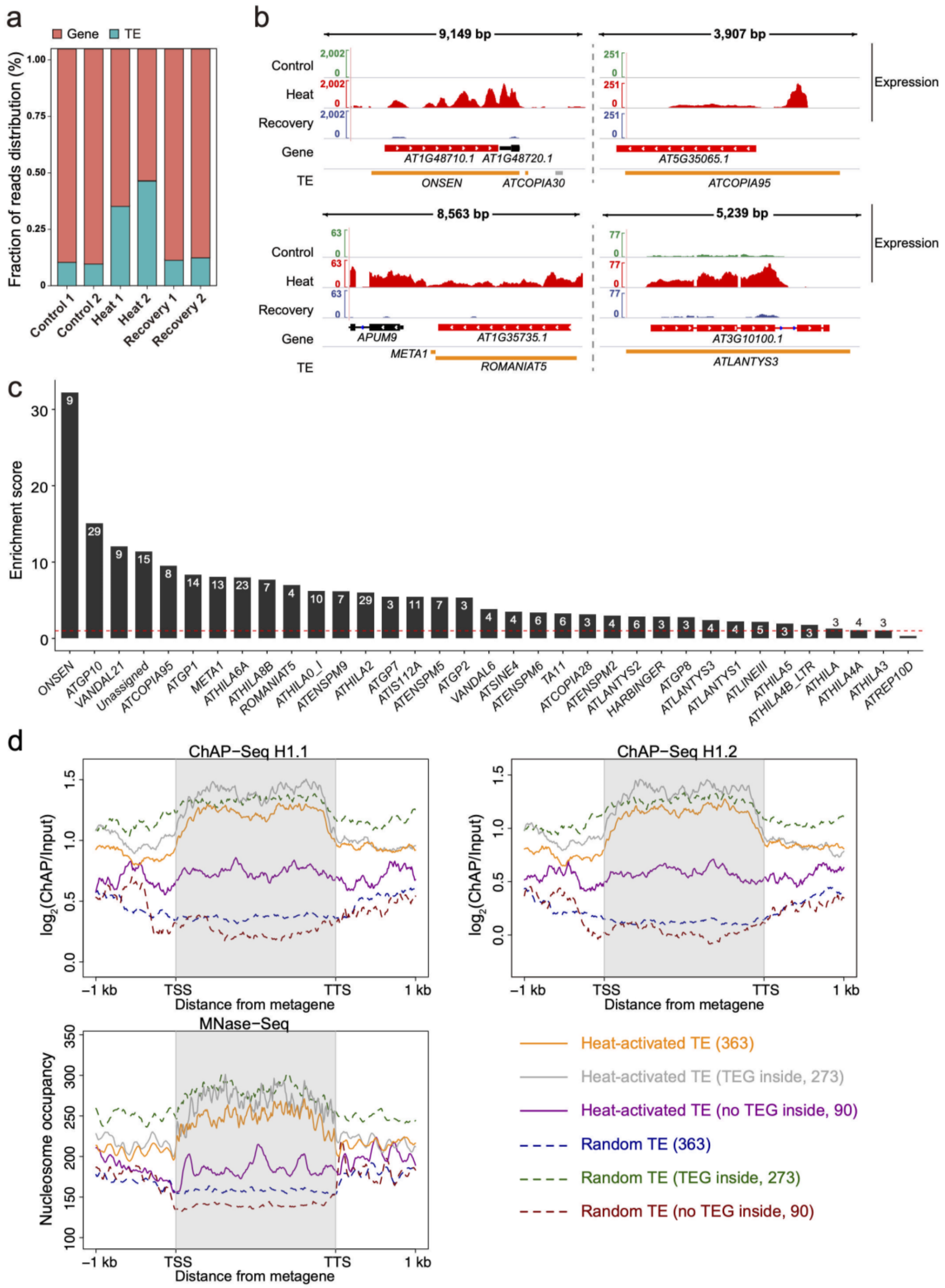

a
Gene
TE
Fraction of reads distribution (%)
Control 1
Control 2
Heat 1
Heat 2
Recovery 1
Recovery 2
b
9,149 bp
3,907 bp
8,563 bp
5,239 bp
Control
Heat
Recovery
Gene
TE
Expression
AT1G48710.1
AT1G48720.1
ONSEN
ATCOPIA30
AT5G35065.1
ATCOPIA95
APUM9
AT1G35735.1
META1
ROMANIAT5
AT3G10100.1
ATLANTYS3
c
Enrichment score
ONSEN
ATGP10
VANDAL21
Unassigned
ATCOPIA95
ATGP1
META1
ATHILA6A
ATHILA6B
ROMANIAT5
ATHILA0_I
ATENSPM9
ATHILA2
ATGP7
ATIS112A
ATENSPM5
ATGP2
VANDAL6
ATSINE4
ATENSPM6
TA11
ATCOPIA28
ATENSPM2
ATLANTYS2
HARBINGER
ATGP8
ATLANTYS3
ATLANTYS1
ATLINEIII
ATHILA5
ATHILA4B_LTR
ATHILA
ATHILA4A
ATHILA3
ATREP10D
d
ChAP−Seq H1.1
ChAP−Seq H1.2
MNase−Seq
log2(ChAP/Input)
Nucleosome occupancy
Distance from metagene
−1 kb
TSS
TTS
1 kb
Heat-activated TE (363)
Heat-activated TE (TEG inside, 273)
Heat-activated TE (no TEG inside, 90)
Random TE (363)
Random TE (TEG inside, 273)
Random TE (no TEG inside, 90)

e

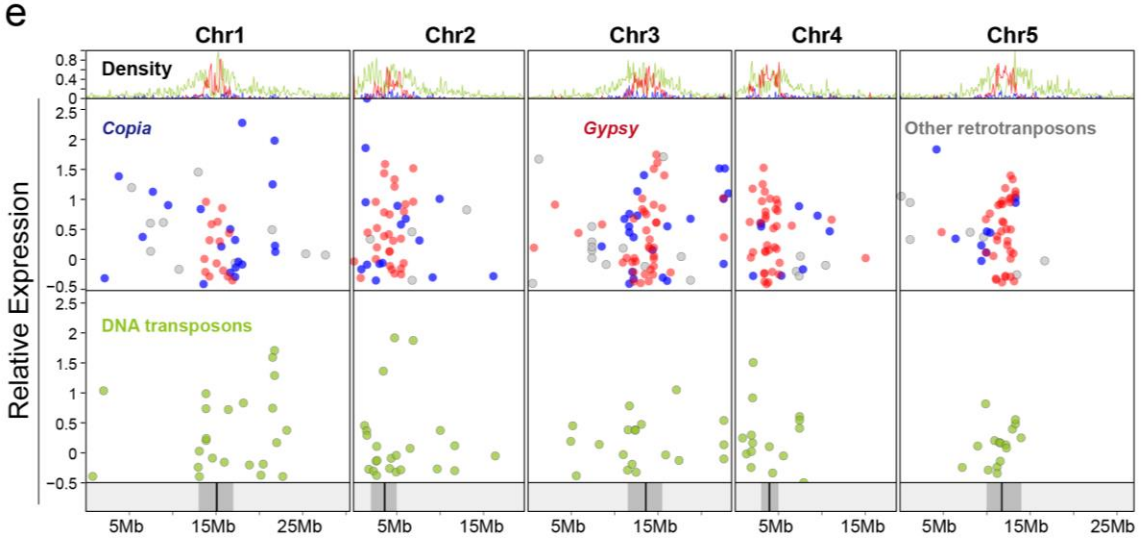

Supplementary Figure 2. RNA-Seq analysis of differentially expressed TEs in different treatments.

a. Stacked bar plot showing the fraction of uniquely mapped reads assigned to genes and TEs in Control, Heat, and Recovery.

b. Genome browser snapshots of relative expression levels of 4 heat-activated TEs from different families in Control, Heat, and Recovery. Snapshot region lengths are marked on the top of each graph. The top 3 tracks show the expression levels identified by RNA-Seq. Gene and TE tracks are shown. Red blocks in gene track are transposon element genes. Yellow blocks in TE track are retrotransposons, while grey blocks are DNA transposons.

c. The enrichment scores of different families of heat-activated TEs. The enrichment score was calculated as the percentage of a certain TE super family in heat-activated TEs divided by the percentage of this TE super family in all TEs in the *Arabidopsis* genome. Numbers marked on each bar indicate the absolute number of heat-activated TEs in that family. The red dotted line marks the point of overrepresentation (enrichment score > 1 if absolute number ≥ 3).

d. ChAP-Seq metagene plot showing the occupancy of heat-activated TEs (all 363 TEs, 273 TEs overlapped with transposable element genes (TEGs), and 90 TEs don't overlap with TEGs) and equal number of random TEs (from total TE and total TE with TEGs or without TEGs) by histones H1.1 and H1.2 under normal conditions. MNase-Seq (from published paper) metagene plot showing the occupancy of nucleosomes under normal

conditions for similar TE groups.

e. Genome-wide distribution of heat-activated TEs. The uppermost track shows the density of *Copia* and *Gyspy* retrotransposon super families and DNA transposons. The 2 tracks in the middle show the distribution of retrotransposons and DNA transposons. Expression difference represents the difference in expression calculated by $\log_{10}$ (Heat $_{CPM}$ - Control $_{CPM}$). TEs belonging to *Copia* and *Gyspy* retrotransposon super families and DNA transposons are colored in blue, red, and green, respectively. Other retrotransposons are colored in gray. The bottom track shows the positions of centromeres (black) and pericentromeres (gray). This panel is related to Fig. 1g.

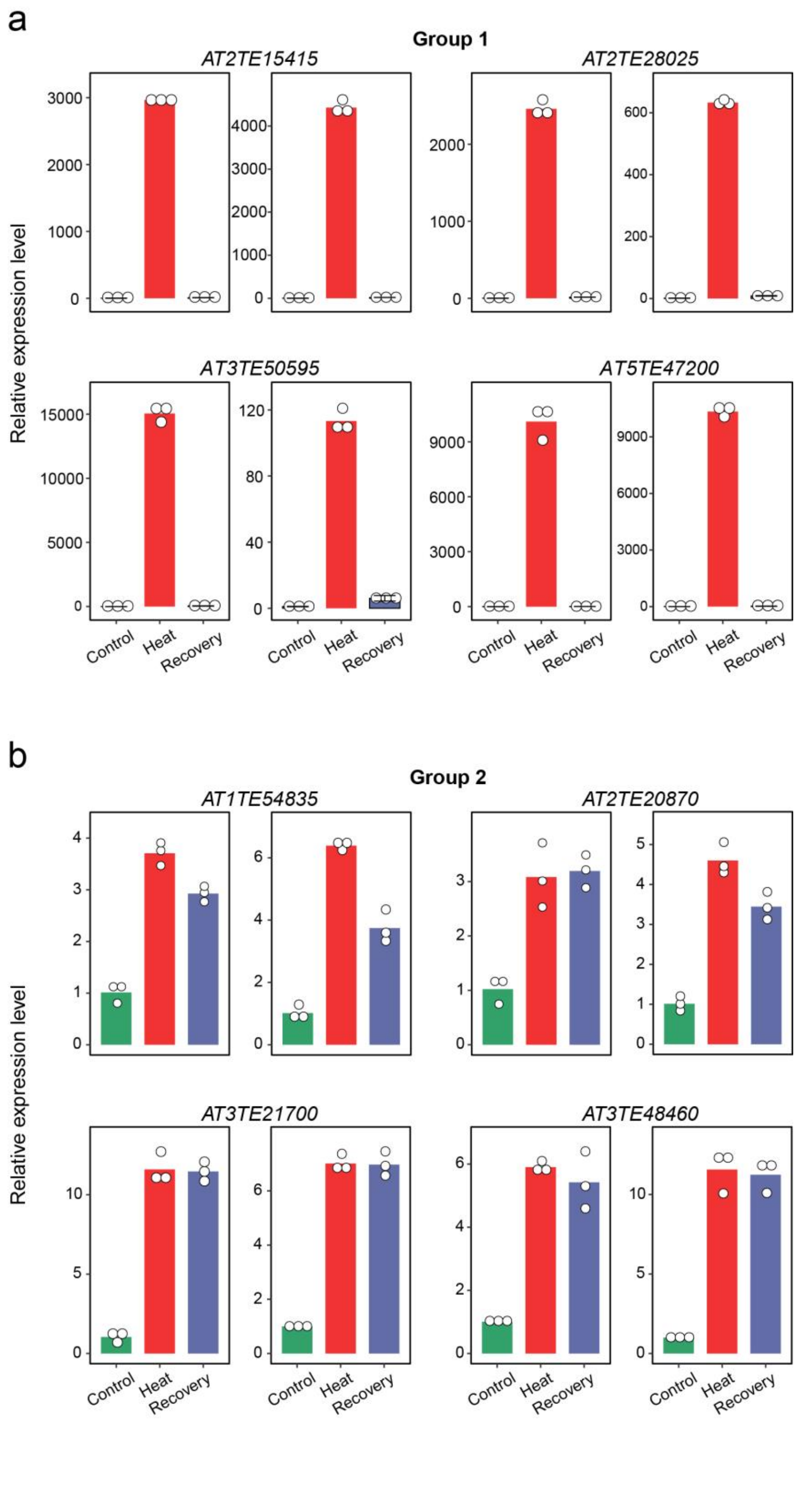
a
Group 1
AT2TE15415
AT2TE28025
AT3TE50595
AT5TE47200
Relative expression level
Control
Heat
Recovery
b
Group 2
AT1TE54835
AT2TE20870
AT3TE21700
AT3TE48460
Relative expression level
Control
Heat
Recovery

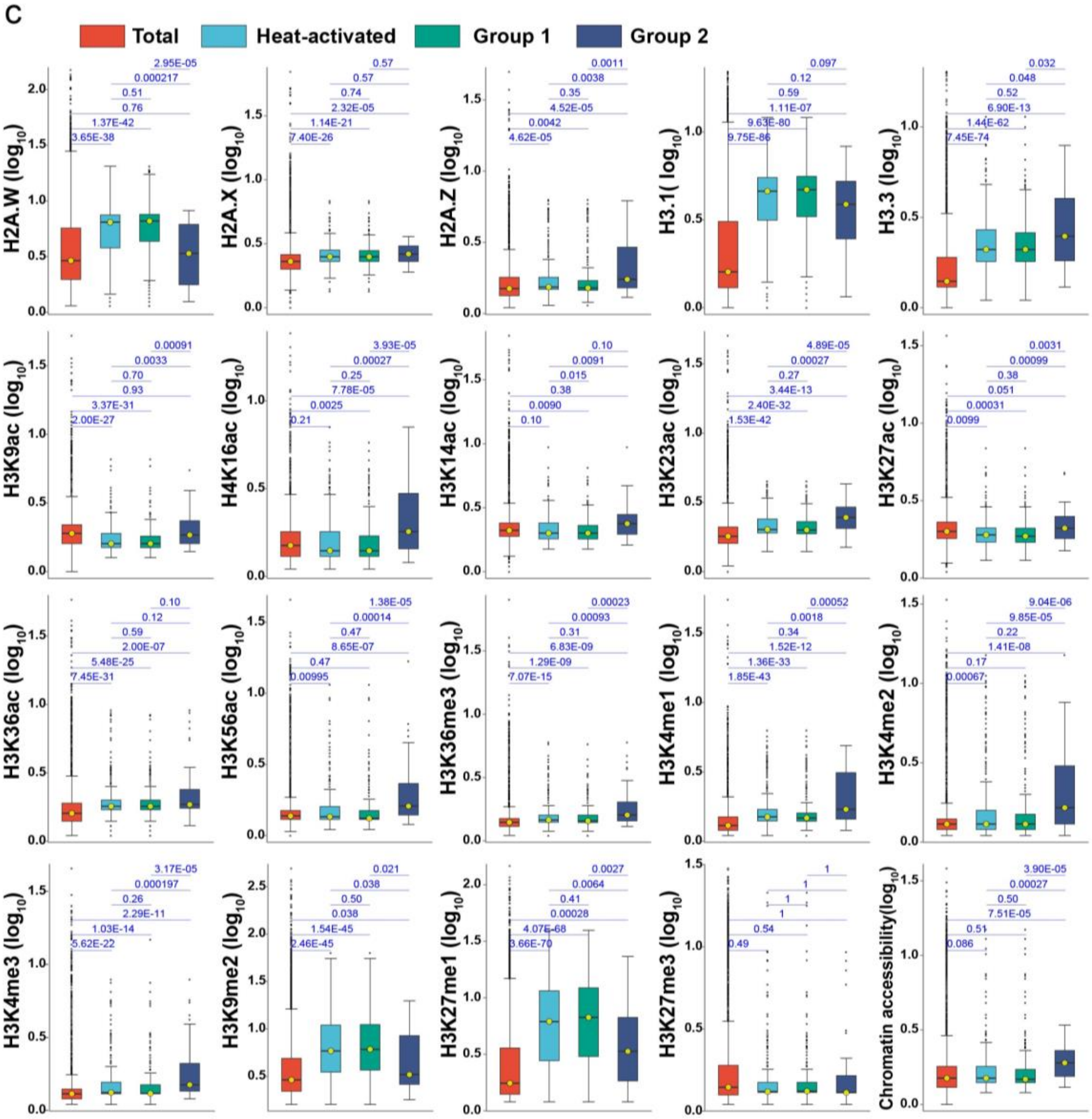

Supplementary Figure 3. Levels of epigenetic modifications in heat-activated TEs and total TEs.

a-b. Expression dynamics of randomly selected Group 1 (a) and Group 2 (b) TEs as determined by RT-qPCR with 2 biological replicates. The expression value of each replicate was normalized to the expression level of Control. Circles denote relative expression values. Bars indicate mean values of three technical replicates in a biological replicate.

c. Box plots showing levels of epigenetic modifications in heat-activated TEs and total TEs in *Arabidopsis*. Pairwise two-sided Mann–Whitney U test *p*-values are shown in blue, and the *p-values* are adjusted by the Bonferroni–Holm method for multiple comparisons.

Heat-activated TEs include Group 1 and Group 2 TEs. For each box plot, center lines indicate the medians; boxes show the 25th and 75th percentiles; whiskers extend to the minimum and maximum. Source data of Supplementary Figure 3a and 3b are provided as a Source Data file.

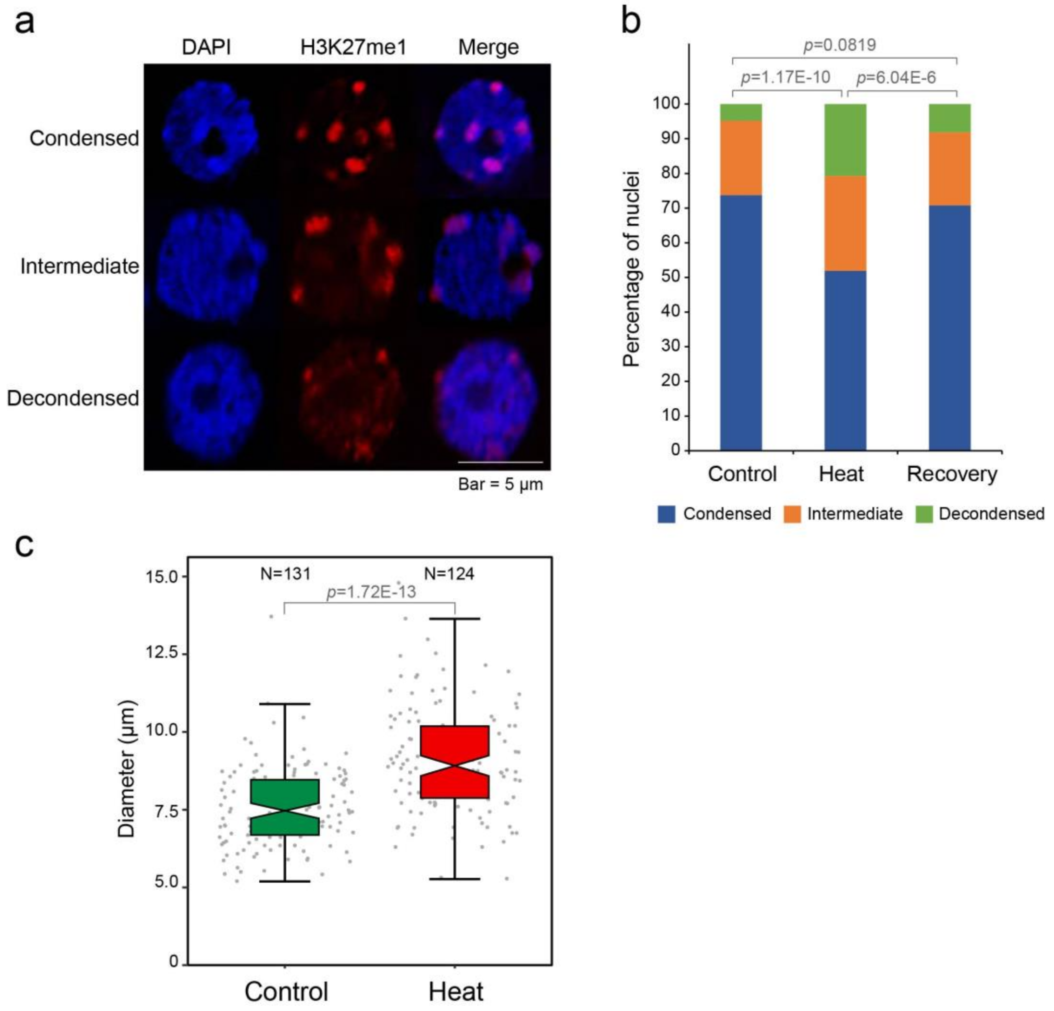

Supplementary Figure 4. Heterochromatin decondensation in different treatments.

a. Examples of 3 types of chromocenters: condensed, intermediate, and decondensed, which differ in their heterochromatin status. Nuclei were stained with DAPI and the H3K27me1 antibody.

b. Percentages of condensed, intermediate, and decondensed chromocenters in Control, Heat, and Recovery nuclei (n = 300). Significant differences between 2 groups are determined by two-sided Fisher's exact test, with *p-values* adjusted by the false discovery rate method for multiple comparisons. Exact *p-values* are shown above the bars.

c. Nuclear size distribution in Control and Heat. *p-value* was determined by the two-sided Mann–Whitney U test. For each box plot, center lines indicate the medians; boxes show the 25th and 75th percentiles; whiskers extend to the minimum and maximum. Source data underlying Supplementary Figure 4b and 4c are provided as a Source Data file.

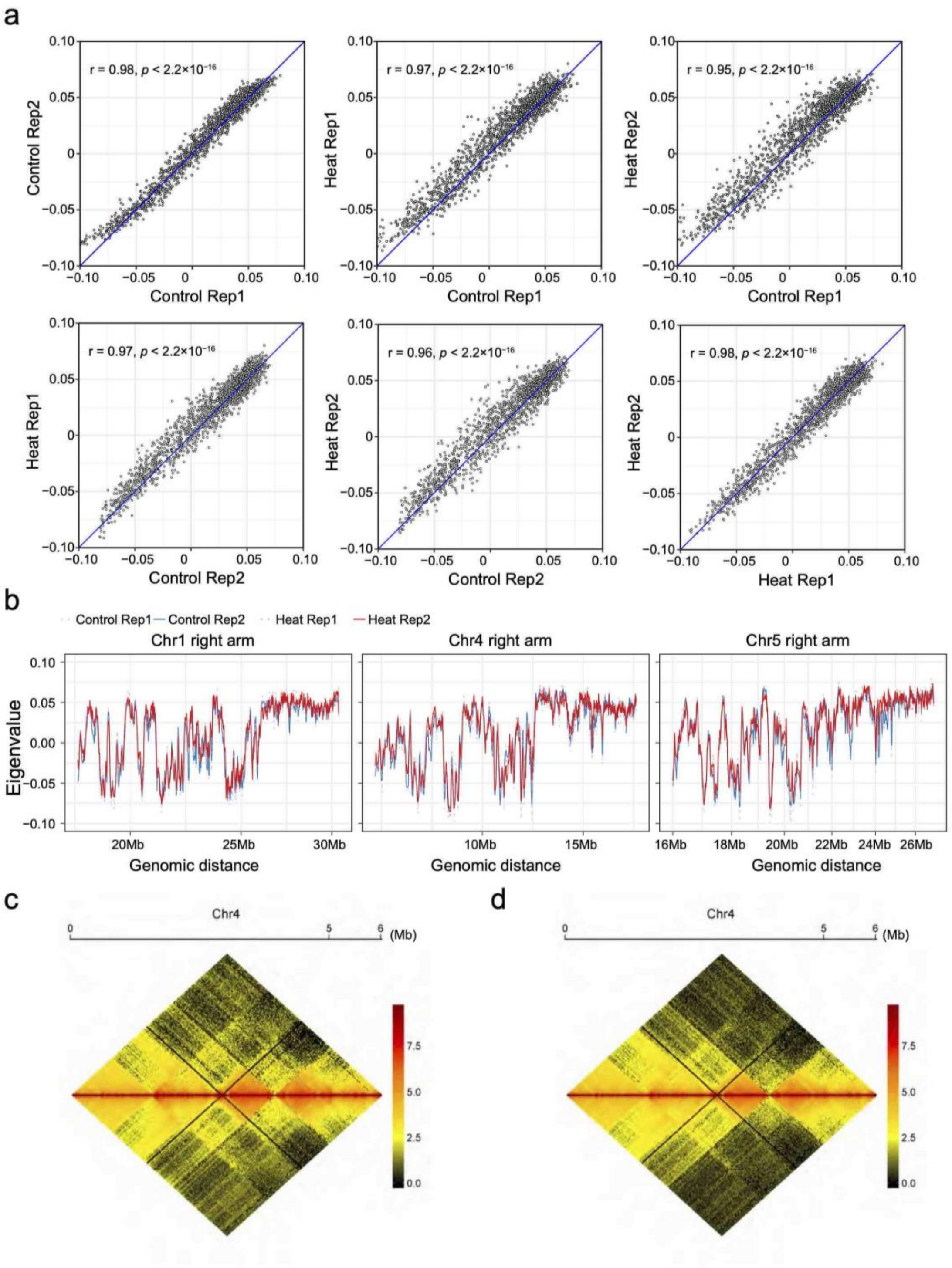

Supplementary Figure 5. Reproducibility of 2 Hi-C biological replicates.

a. Scatterplot showing all possible combinations of the genome-wide correlations between eigenvalues of the PC1 calculated using 2 biological replicates of Hi-C data from Control and Heat at 20 kb resolution. *p-value* are generated by two-sided pearson correlation test

.

b. The eigenvectors of the PC1 (which accounts for the greatest proportion of the total variance) for the right arms of chromosomes 1, 4, and 5 at 20 kb resolution. They were calculated using 2 biological replicates of Control and Heat Hi-C data.

c-d. Hi-C chromatin interaction heatmaps comparing interaction frequencies at the first 6 megabases of chromosome 4 in 2 biological replicates of Control (c) and Heat (d) at 20 kb resolution.

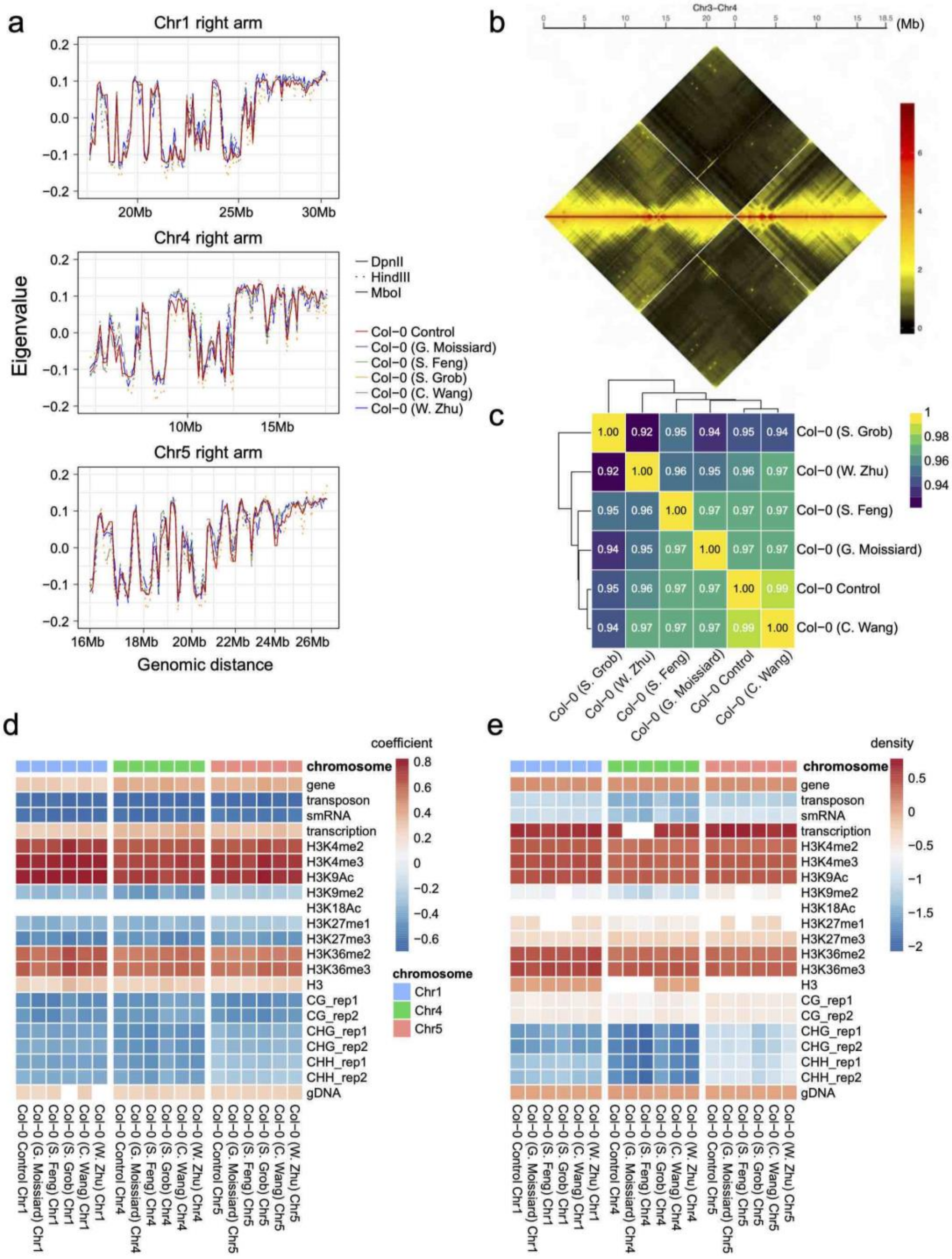

a
Chr1 right arm
Chr4 right arm
Chr5 right arm
Eigenvalue
Genomic distance
20Mb
25Mb
30Mb
10Mb
15Mb
16Mb
18Mb
20Mb
22Mb
24Mb
26Mb
DpnII
HindIII
MboI
Col−0 Control
Col−0 (G. Moissiard)
Col−0 (S. Feng)
Col−0 (S. Grob)
Col−0 (C. Wang)
Col−0 (W. Zhu)
b
Chr3−Chr4
(Mb)
c
Col−0 (S. Grob)
Col−0 (W. Zhu)
Col−0 (S. Feng)
Col−0 (G. Moissiard)
Col−0 Control
Col−0 (C. Wang)
d
coefficient
e
density
chromosome
gene
transposon
smRNA
transcription
H3K4me2
H3K4me3
H3K9Ac
H3K9me2
H3K18Ac
H3K27me1
H3K27me3
H3K36me2
H3K36me3
H3
CG_rep1
CG_rep2
CHG_rep1
CHG_rep2
CHH_rep1
CHH_rep2
gDNA
Chr1
Chr4
Chr5

Supplementary Figure 6. Comparisons of our Hi-C datasets with previously published Hi-C datasets.

a. Graphs of the eigenvectors of the PC1 calculated using our and previously published Hi-C datasets for the right arms of chromosomes 1, 4, and 5 at 100 kb resolution.

b. Hi-C chromatin interaction heatmap at 100 kb resolution comparing interaction frequencies within and between chromosomes 3 and 4 in Control and Col-0 (Wang *et al*., 2015.).

c. Pearson correlation coefficients calculated from the medians of correlations between all the individual bins of the interaction matrices from published and in-house generated Hi-C interaction data at 100 kb resolution.

d. Pearson correlation coefficients between the eigenvectors of the PC1 calculated using published and in-house generated Hi-C interaction datasets and epigenomic and genomic features in the right arms of chromosomes 1, 4, and 5 at 100 kb resolution.

e. The significant enrichment and depletion of epigenomic and genomic features between regions with positive eigenvalues and negative eigenvalues in the right arms of chromosomes 1, 4, and 5 at 100 kb resolution.

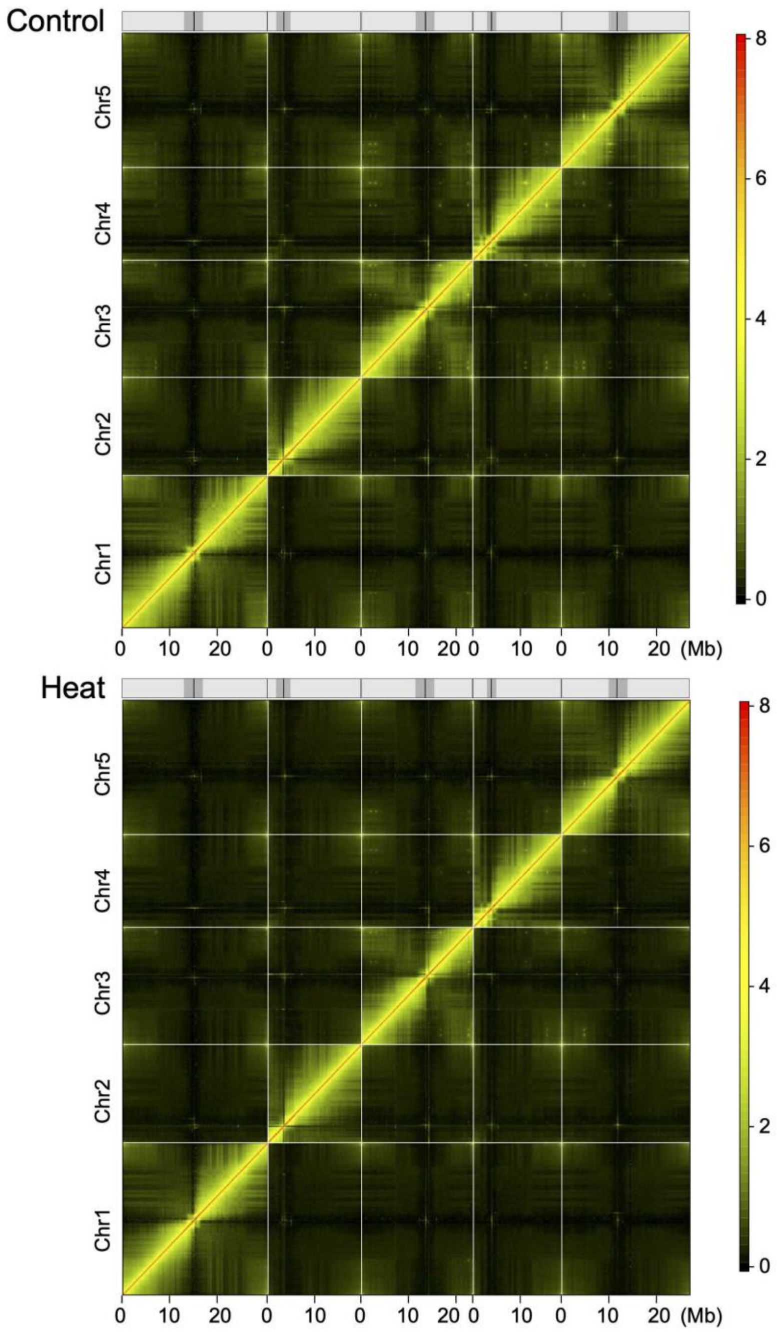

Supplementary Figure 7. Hi-C heatmaps at 100 kb resolution for all chromosomes in Control and Heat.

The top heatmap shows chromatin interaction patterns in Control and the bottom heatmap shows chromatin interaction patterns in Heat. Both heatmaps use the same color scheme. Diagonal line values are set to zero. The top track of each heatmap shows the positions of centromeres (black) and pericentromeres (gray).

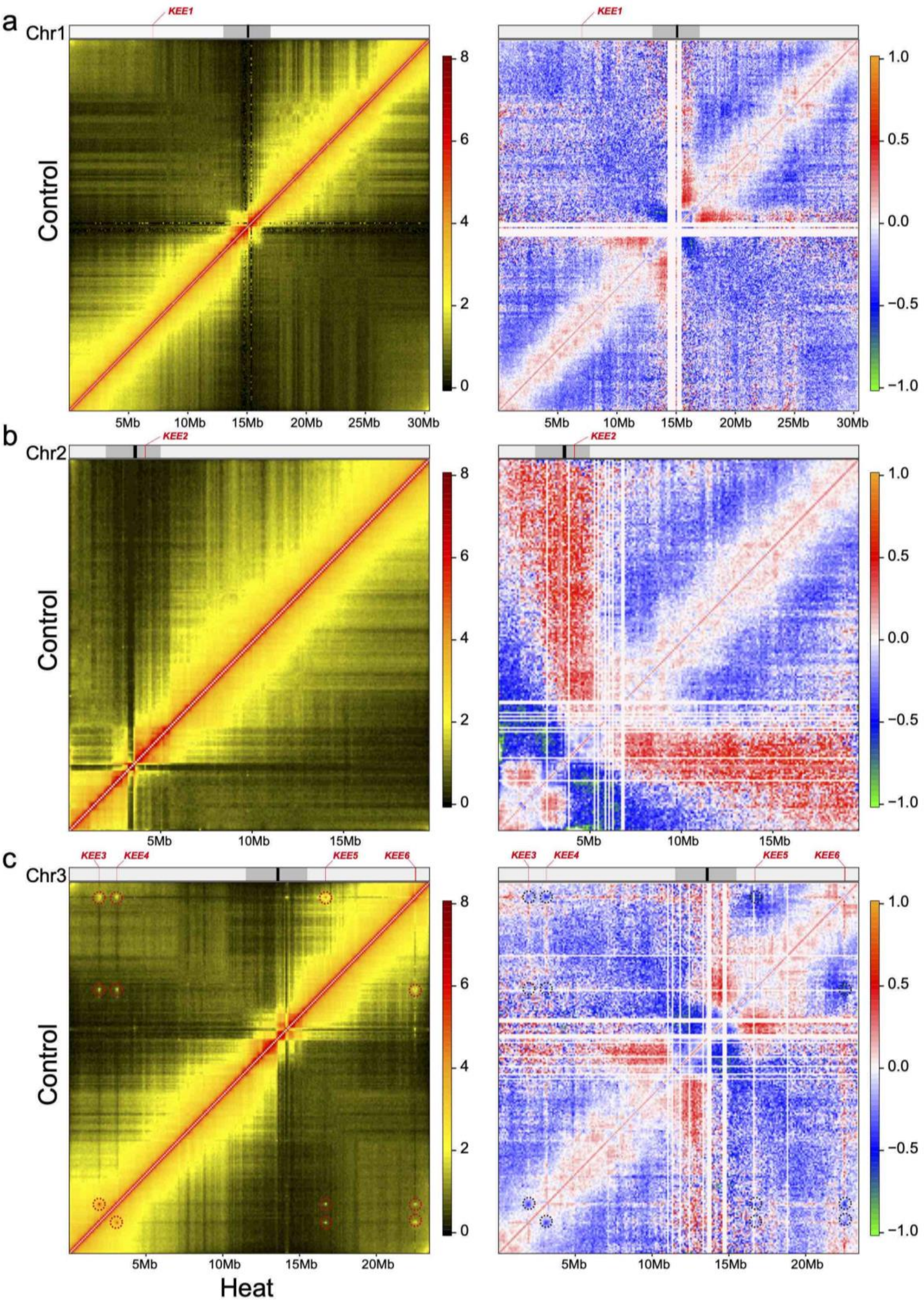
a
Chr1
KEE1
Control
5Mb
10Mb
15Mb
20Mb
25Mb
30Mb
8
6
4
2
0
1.0
0.5
0.0
−0.5
−1.0
b
Chr2
KEE2
Control
5Mb
10Mb
15Mb
c
Chr3
KEE3
KEE4
KEE5
KEE6
Control
5Mb
10Mb
15Mb
20Mb
Heat

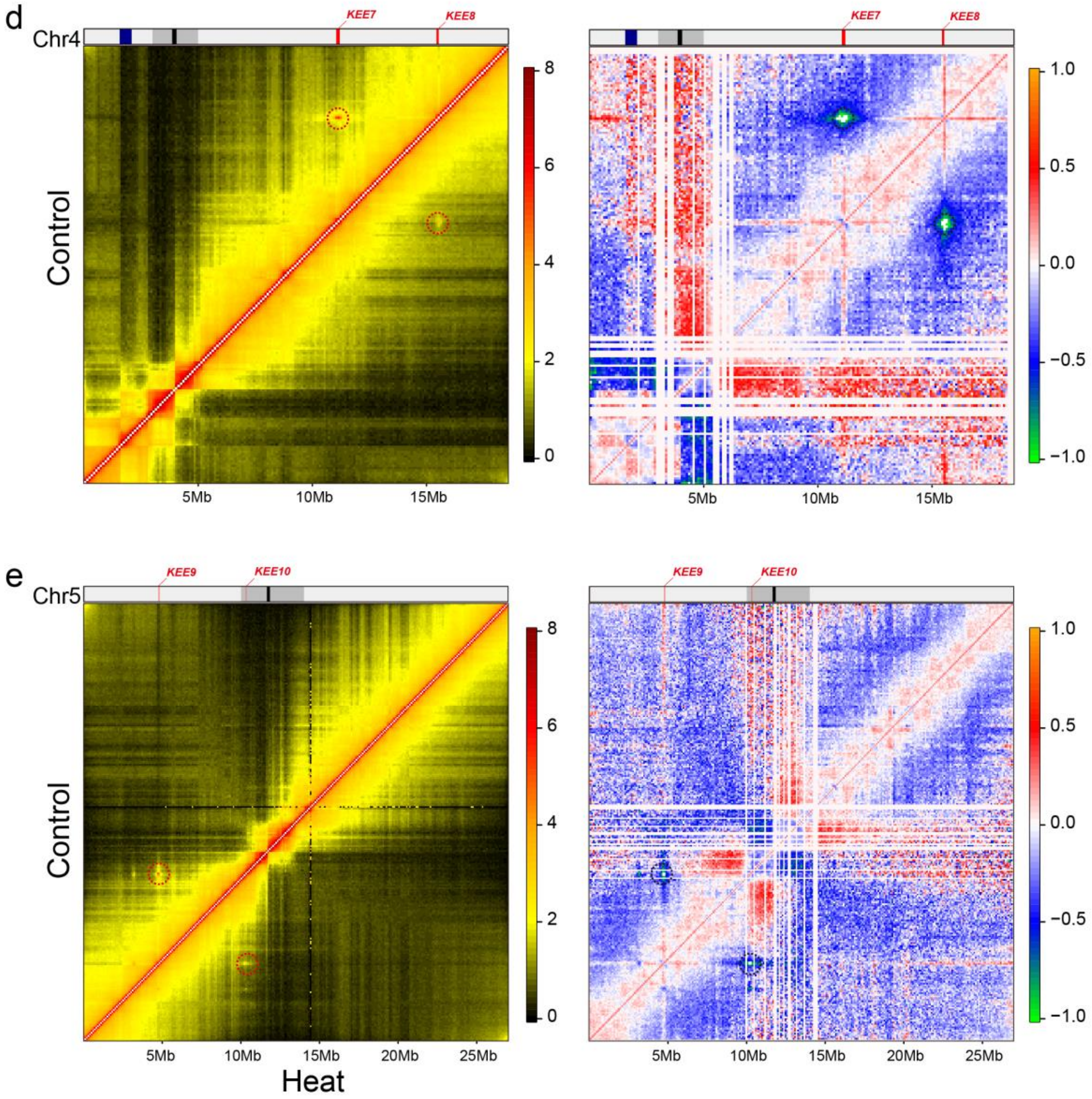
d
Chr4
KEE7
KEE8
Control
8
6
4
2
0
1.0
0.5
0.0
−0.5
−1.0
5Mb
10Mb
15Mb
e
Chr5
KEE9
KEE10
Control
Heat
5Mb
10Mb
15Mb
20Mb
25Mb

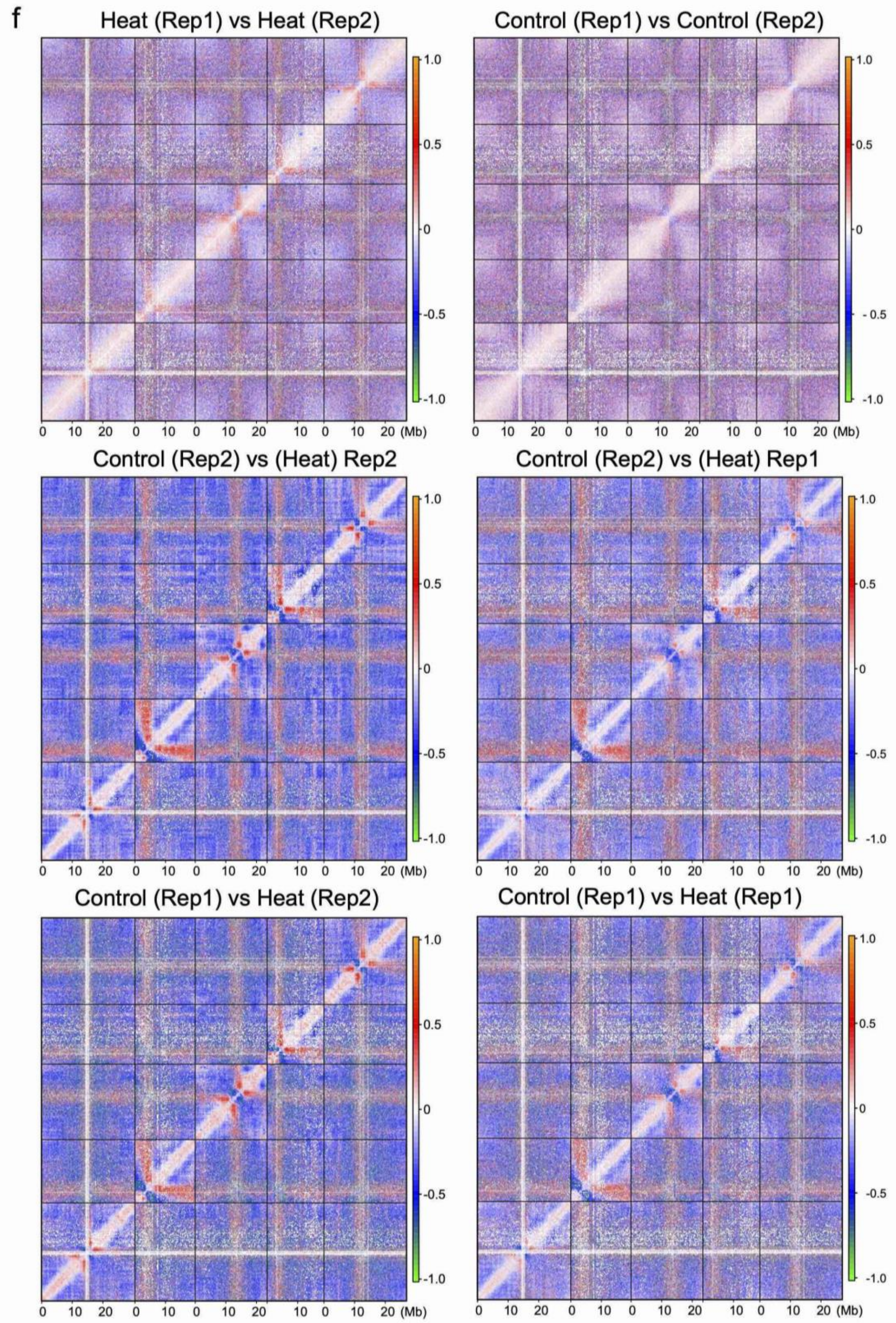
f
Heat (Rep1) vs Heat (Rep2)
Control (Rep1) vs Control (Rep2)
Control (Rep2) vs (Heat) Rep2
Control (Rep2) vs (Heat) Rep1
Control (Rep1) vs Heat (Rep2)
Control (Rep1) vs Heat (Rep1)
1.0
0.5
0
-0.5
-1.0
0 10 20 0 10 0 10 20 10 0 10 20 (Mb)

Supplementary Figure 8. Hi-C Heatmaps at 100 kb resolution for each chromosome in Control and Heat.

a-e. Left: The heatmap at the top left corner shows chromatin interaction patterns within a chromosome in control and the heatmap at the bottom right corner shows chromatin interaction patterns within a chromosome in Heat. Both heatmaps use the same color scheme and are at 100 kb resolution. Diagonal line values are set to zero. Right: The heatmap shows relative differences of interaction frequencies within a chromosome in Control and Heat at 100 kb resolution. The top track of each heatmap shows the positions of centromeres (black), pericentromeres (gray), the knob *hk4s* (blue), and *KEE*s (red). *KEEs* are labeled by red and black dashed circles in chromatin interaction heatmaps and relative differences heatmaps, respectively.

f. Heatmaps showing relative differences of interaction frequencies between Heat Rep1 and Heat Rep2, Control Rep1 and Control Rep2, Control Rep2 and Heat Rep2, Control Rep2 and Heat Rep1, Control Rep1 and Heat Rep2, and Control Rep1 and Heat Rep1.

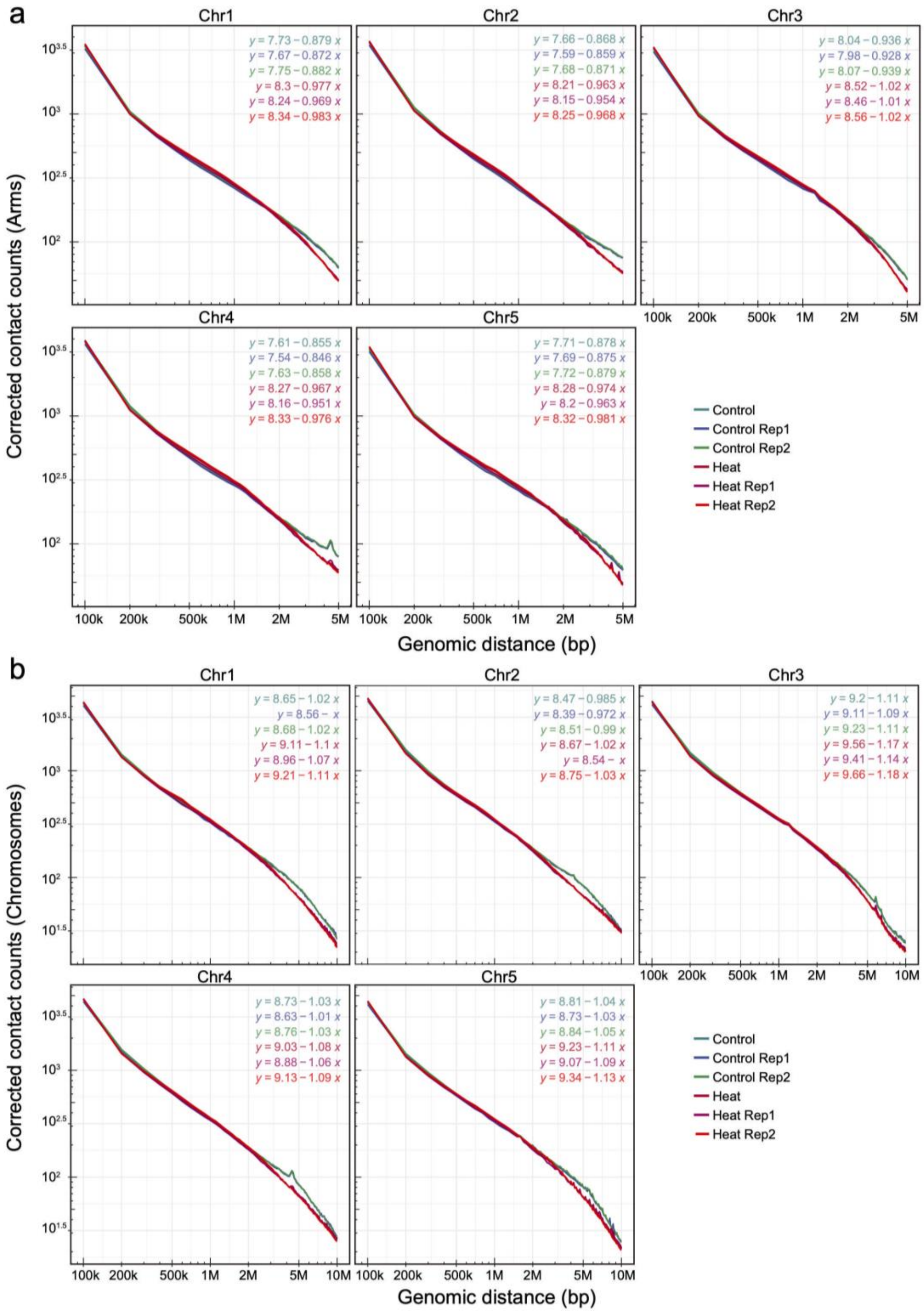

a
Chr1
y = 7.73 − 0.879 x
y = 7.67 − 0.872 x
y = 7.75 − 0.882 x
y = 8.3 − 0.977 x
y = 8.24 − 0.969 x
y = 8.34 − 0.983 x
Chr2
y = 7.66 − 0.868 x
y = 7.59 − 0.859 x
y = 7.68 − 0.871 x
y = 8.21 − 0.963 x
y = 8.15 − 0.954 x
y = 8.25 − 0.968 x
Chr3
y = 8.04 − 0.936 x
y = 7.98 − 0.928 x
y = 8.07 − 0.939 x
y = 8.52 − 1.02 x
y = 8.46 − 1.01 x
y = 8.56 − 1.02 x
Chr4
y = 7.61 − 0.855 x
y = 7.54 − 0.846 x
y = 7.63 − 0.858 x
y = 8.27 − 0.967 x
y = 8.16 − 0.951 x
y = 8.33 − 0.976 x
Chr5
y = 7.71 − 0.878 x
y = 7.69 − 0.875 x
y = 7.72 − 0.879 x
y = 8.28 − 0.974 x
y = 8.2 − 0.963 x
y = 8.32 − 0.981 x
Corrected contact counts (Arms)
Genomic distance (bp)
100k 200k 500k 1M 2M 5M
Control
Control Rep1
Control Rep2
Heat
Heat Rep1
Heat Rep2
b
Chr1
y = 8.65 − 1.02 x
y = 8.56 − x
y = 8.68 − 1.02 x
y = 9.11 − 1.1 x
y = 8.96 − 1.07 x
y = 9.21 − 1.11 x
Chr2
y = 8.47 − 0.985 x
y = 8.39 − 0.972 x
y = 8.51 − 0.99 x
y = 8.67 − 1.02 x
y = 8.54 − x
y = 8.75 − 1.03 x
Chr3
y = 9.2 − 1.11 x
y = 9.11 − 1.09 x
y = 9.23 − 1.11 x
y = 9.56 − 1.17 x
y = 9.41 − 1.14 x
y = 9.66 − 1.18 x
Chr4
y = 8.73 − 1.03 x
y = 8.63 − 1.01 x
y = 8.76 − 1.03 x
y = 9.03 − 1.08 x
y = 8.88 − 1.06 x
y = 9.13 − 1.09 x
Chr5
y = 8.81 − 1.04 x
y = 8.73 − 1.03 x
y = 8.84 − 1.05 x
y = 9.23 − 1.11 x
y = 9.07 − 1.09 x
y = 9.34 − 1.13 x
Corrected contact counts (Chromosomes)
Genomic distance (bp)
100k 200k 500k 1M 2M 5M 10M
Control
Control Rep1
Control Rep2
Heat
Heat Rep1
Heat Rep2

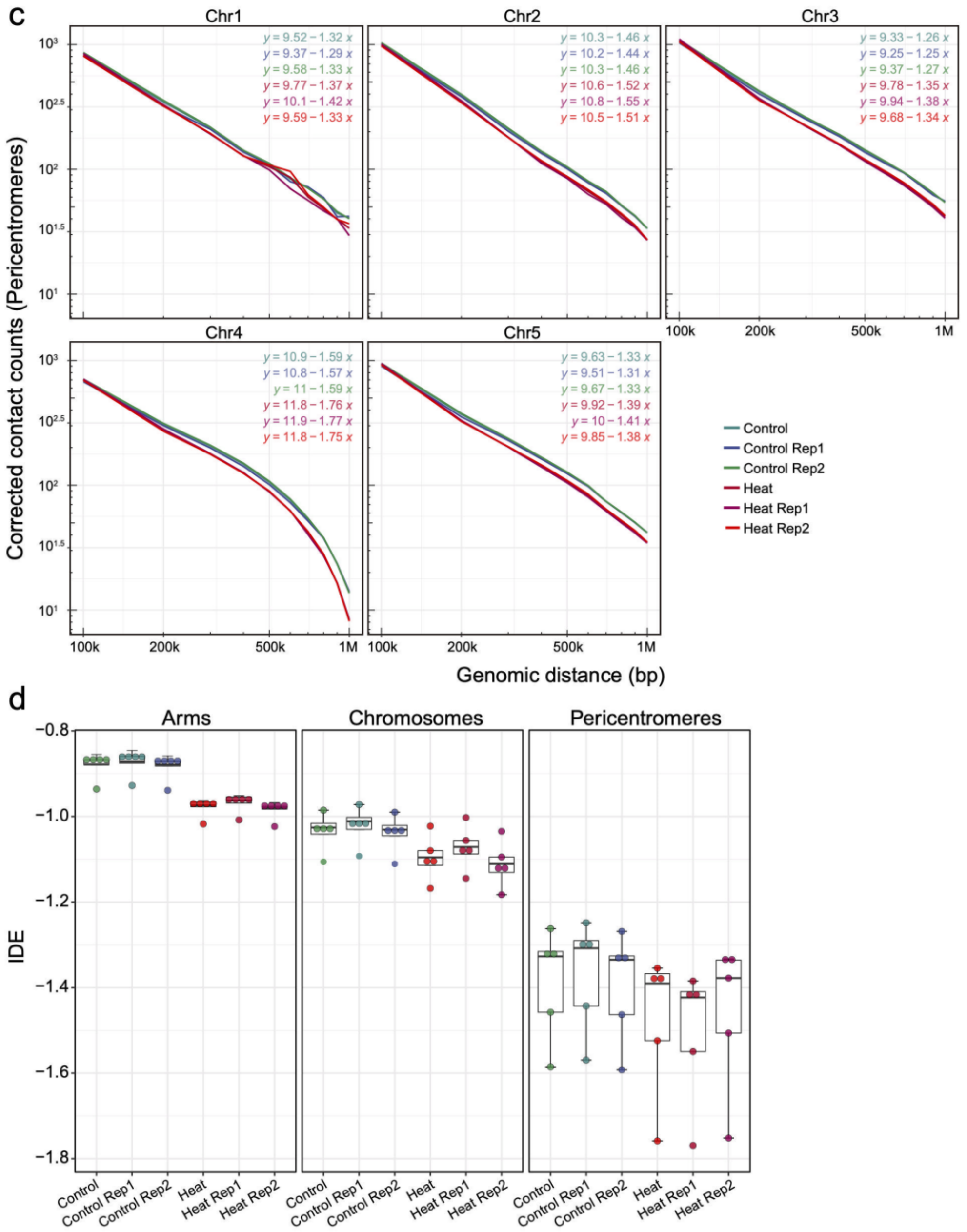

Supplementary Figure 9. Scaling plots of interaction frequencies against increasing genomic distance at 100 kb resolution.

a-c. Scaling plots of interaction frequencies against increasing genomic distance for all chromosome arms (a), chromosomes (b), and pericentromeres (c) in Control, Heat, and each of their biological replicates. The genomic bin size is 100 kb.

d. The distribution of average interaction decay exponents (IDEs) of chromosome arms, chromosomes, and pericentromeres in Control, Heat, and each of their biological replicates. For each box plot, center lines indicate the medians; boxes show the 25th and

75th percentiles; whiskers extend to the minimum and maximum. Source data underlying Supplementary Figure 9d are provided as a Source Data file.

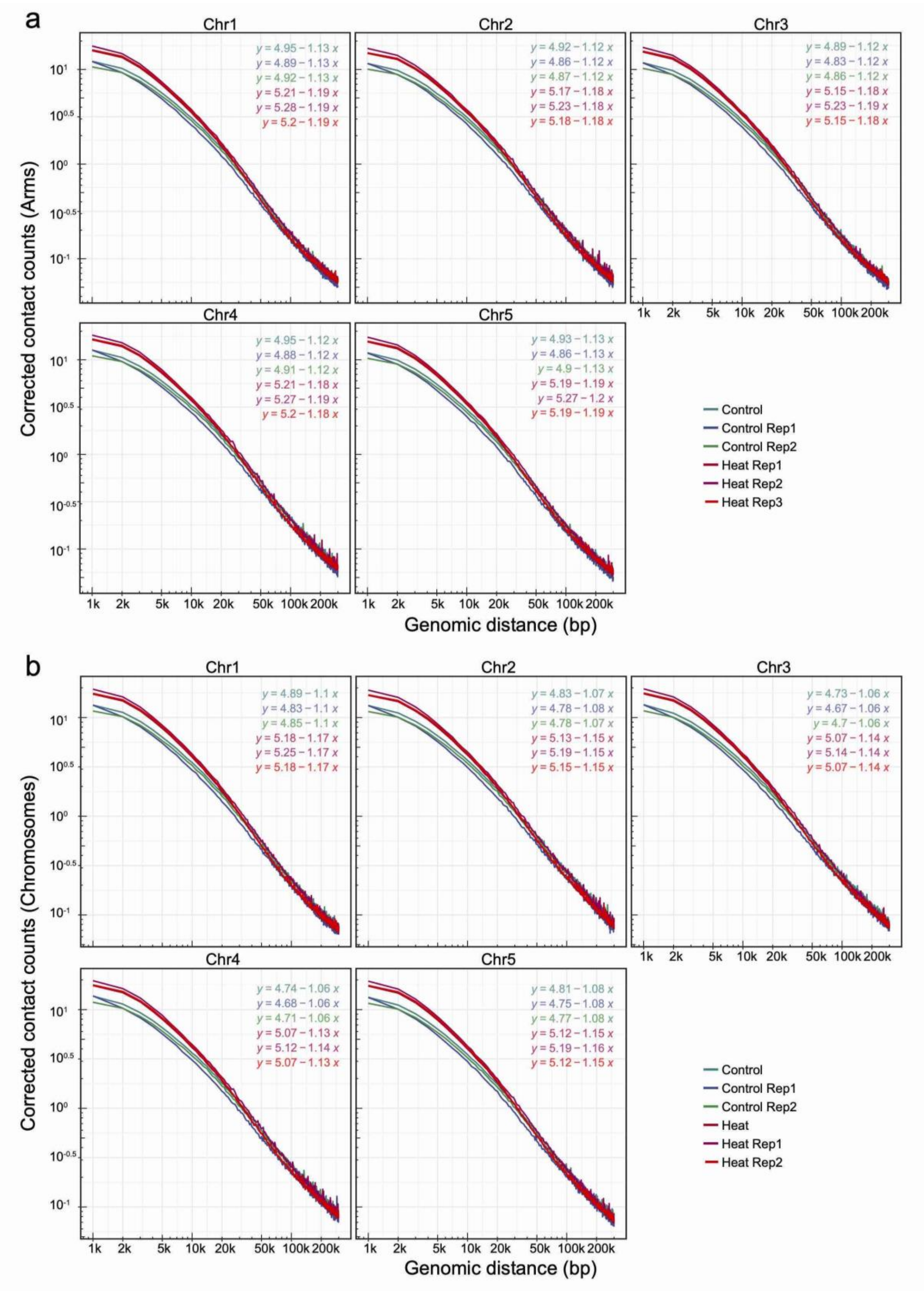
a
Chr1
y = 4.95 − 1.13 x
y = 4.89 − 1.13 x
y = 4.92 − 1.13 x
y = 5.21 − 1.19 x
y = 5.28 − 1.19 x
y = 5.2 − 1.19 x
Chr2
y = 4.92 − 1.12 x
y = 4.86 − 1.12 x
y = 4.87 − 1.12 x
y = 5.17 − 1.18 x
y = 5.23 − 1.18 x
y = 5.18 − 1.18 x
Chr3
y = 4.89 − 1.12 x
y = 4.83 − 1.12 x
y = 4.86 − 1.12 x
y = 5.15 − 1.18 x
y = 5.23 − 1.19 x
y = 5.15 − 1.18 x
Chr4
y = 4.95 − 1.12 x
y = 4.88 − 1.12 x
y = 4.91 − 1.12 x
y = 5.21 − 1.18 x
y = 5.27 − 1.19 x
y = 5.2 − 1.18 x
Chr5
y = 4.93 − 1.13 x
y = 4.86 − 1.13 x
y = 4.9 − 1.13 x
y = 5.19 − 1.19 x
y = 5.27 − 1.2 x
y = 5.19 − 1.19 x
Corrected contact counts (Arms)
Genomic distance (bp)
Control
Control Rep1
Control Rep2
Heat Rep1
Heat Rep2
Heat Rep3
b
Chr1
y = 4.89 − 1.1 x
y = 4.83 − 1.1 x
y = 4.85 − 1.1 x
y = 5.18 − 1.17 x
y = 5.25 − 1.17 x
y = 5.18 − 1.17 x
Chr2
y = 4.83 − 1.07 x
y = 4.78 − 1.08 x
y = 4.78 − 1.07 x
y = 5.13 − 1.15 x
y = 5.19 − 1.15 x
y = 5.15 − 1.15 x
Chr3
y = 4.73 − 1.06 x
y = 4.67 − 1.06 x
y = 4.7 − 1.06 x
y = 5.07 − 1.14 x
y = 5.14 − 1.14 x
y = 5.07 − 1.14 x
Chr4
y = 4.74 − 1.06 x
y = 4.68 − 1.06 x
y = 4.71 − 1.06 x
y = 5.07 − 1.13 x
y = 5.12 − 1.14 x
y = 5.07 − 1.13 x
Chr5
y = 4.81 − 1.08 x
y = 4.75 − 1.08 x
y = 4.77 − 1.08 x
y = 5.12 − 1.15 x
y = 5.19 − 1.16 x
y = 5.12 − 1.15 x
Corrected contact counts (Chromosomes)
Genomic distance (bp)
Control
Control Rep1
Control Rep2
Heat
Heat Rep1
Heat Rep2

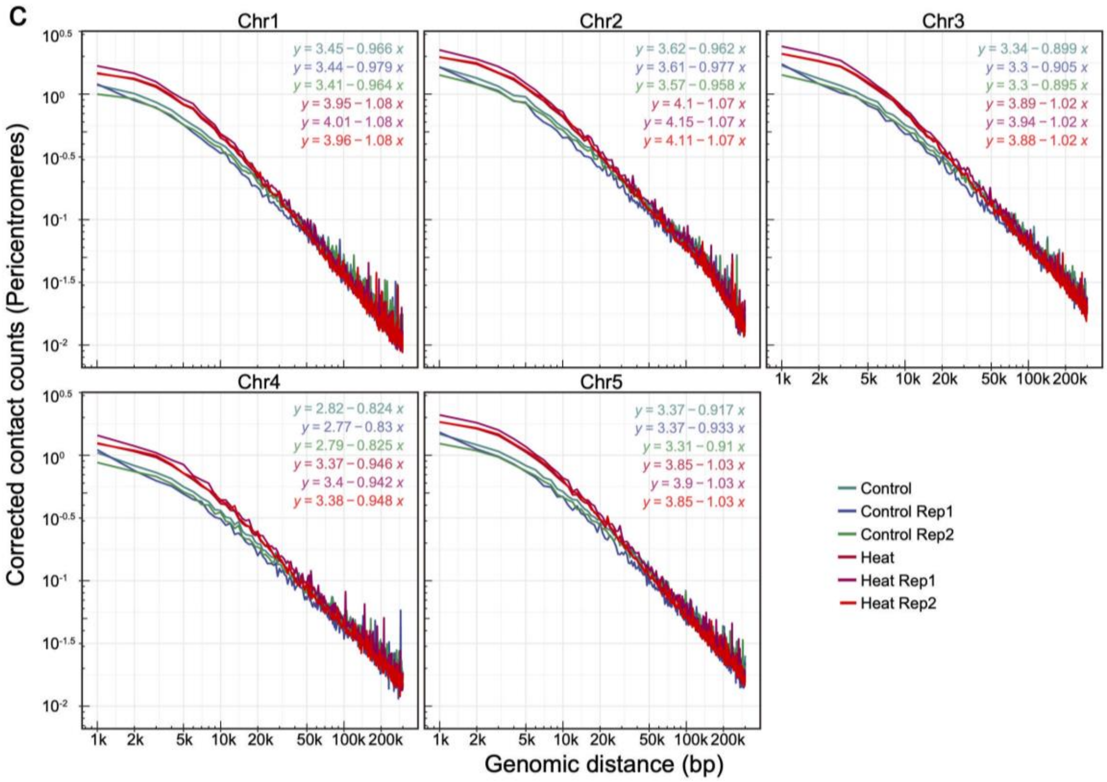

Supplementary Figure 10. Scaling plots of interaction frequencies against increasing genomic distance at 1 kb resolution.

a-c. Scaling plots of interaction frequencies against increasing genomic distance for all chromosome arms (a), chromosomes (b), and pericentromeres (c) in Control, Heat, and each of their biological replicates. The genomic bin size is 1 kb.

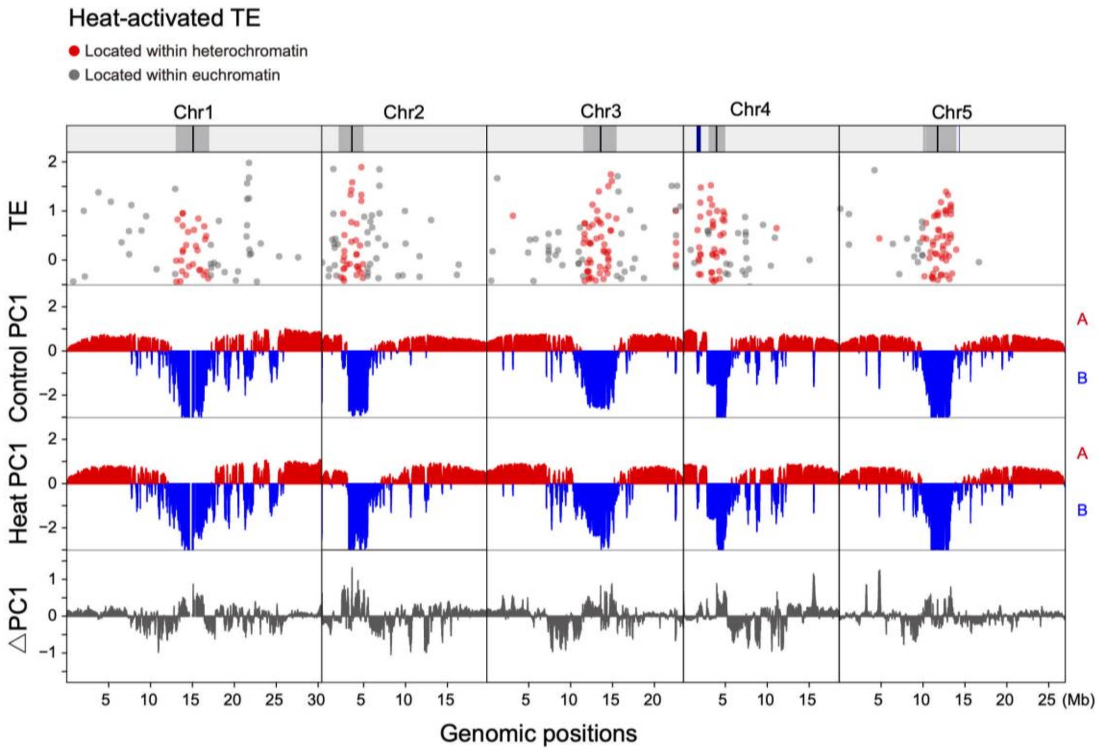

Supplementary Figure 11. Genome-wide distribution of heat-activated TEs and changes in chromatin compartments.

The lower panel shows the positions and differential expression of heat-activated TEs within all chromosomes, PC1 values in Control condition, PC1 values in Heat condition, and ΔPC1 values ($PC1_{Heat} - PC1_{Control}$) horizontally. The positive PC1 values indicate A compartments. The negative PC1 values indicate B compartments.

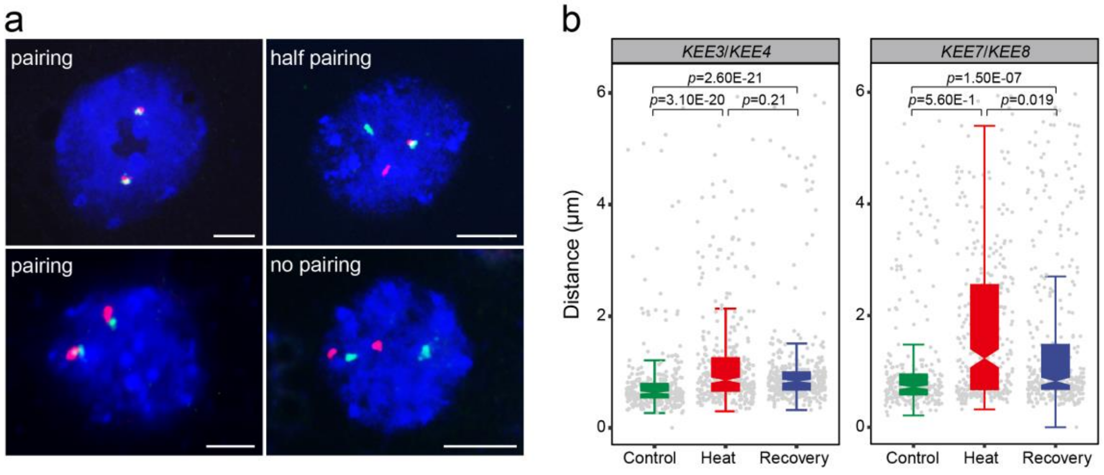

Supplementary Figure 12. Examples of 4 types of interactions between 2 genomic loci in FISH.

a. Bacterial artificial chromosome (BAC) probes were labeled with either biotin or digoxigenin and stained red and green, respectively. Nuclei were counterstained with DAPI. For every 2 BAC clones, 2 complete pairing or proximal events were counted as pairing. One complete pairing event and another separate pair was counted as a half pairing. Two separate pairs were counted as no pairing. Bars = 2 μm.

b. Distance distribution of *KEE3/KEE4* and *KEE7/KEE8* in Control, Heat, and Recovery. Two-sided Mann–Whitney U test *p*-values are shown, and the *p-values* are adjusted by the Bonferroni–Holm method for multiple comparisons. For each box plot, center lines indicate the medians; boxes show the 25th and 75th percentiles; whiskers extend to the minimum and maximum. Source data underlying Supplementary Figure 12b are provided as a Source Data file.

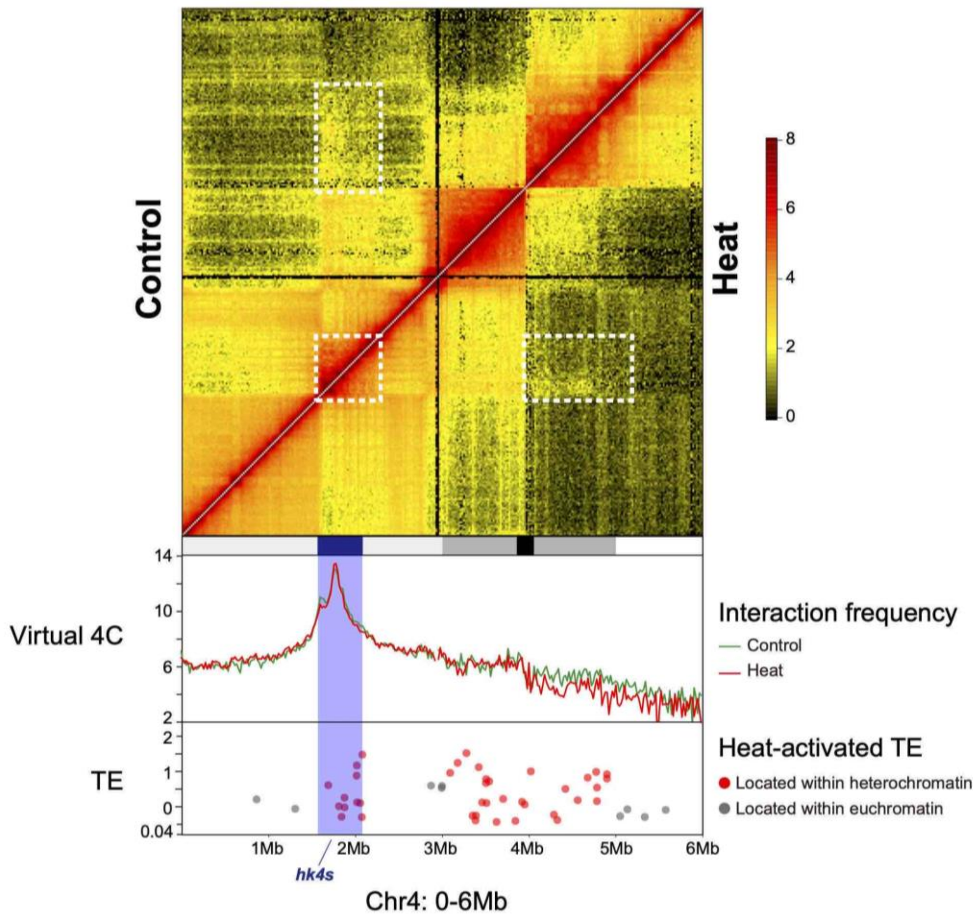

Supplementary Figure 13. Chromatin decondensation within the knob and the mid-range chromosome loop between the knob and the left pericentromere of chromosome 4 under heat stress.

Heatmap showing contact frequencies at the first 6 megabases of chromosome 4 in Control and Heat. The upper- and lower-triangular matrices correspond to Control and Heat, respectively. The values on the diagonal line (interactions between nearby bins) are assigned zero. The square dashed box encloses the interactions within the knob in Control and Heat. The rectangular dashed boxes enclose the interaction frequencies between the knob and the right half of the pericentromere in chromosome 4 in Control and Heat. The track below shows the positions of centromeres (black), pericentromeres (gray), and the knob *hk4s* (blue). The lower panels show the virtual 4C interaction profile from the viewpoint (Chr4:1,760,000-1,780,000) within the knob, the positions and differential expression of heat-activated TEs at the first 6 megabases of chromosome 4. The position of the knob *hk4s* is indicated in violet.

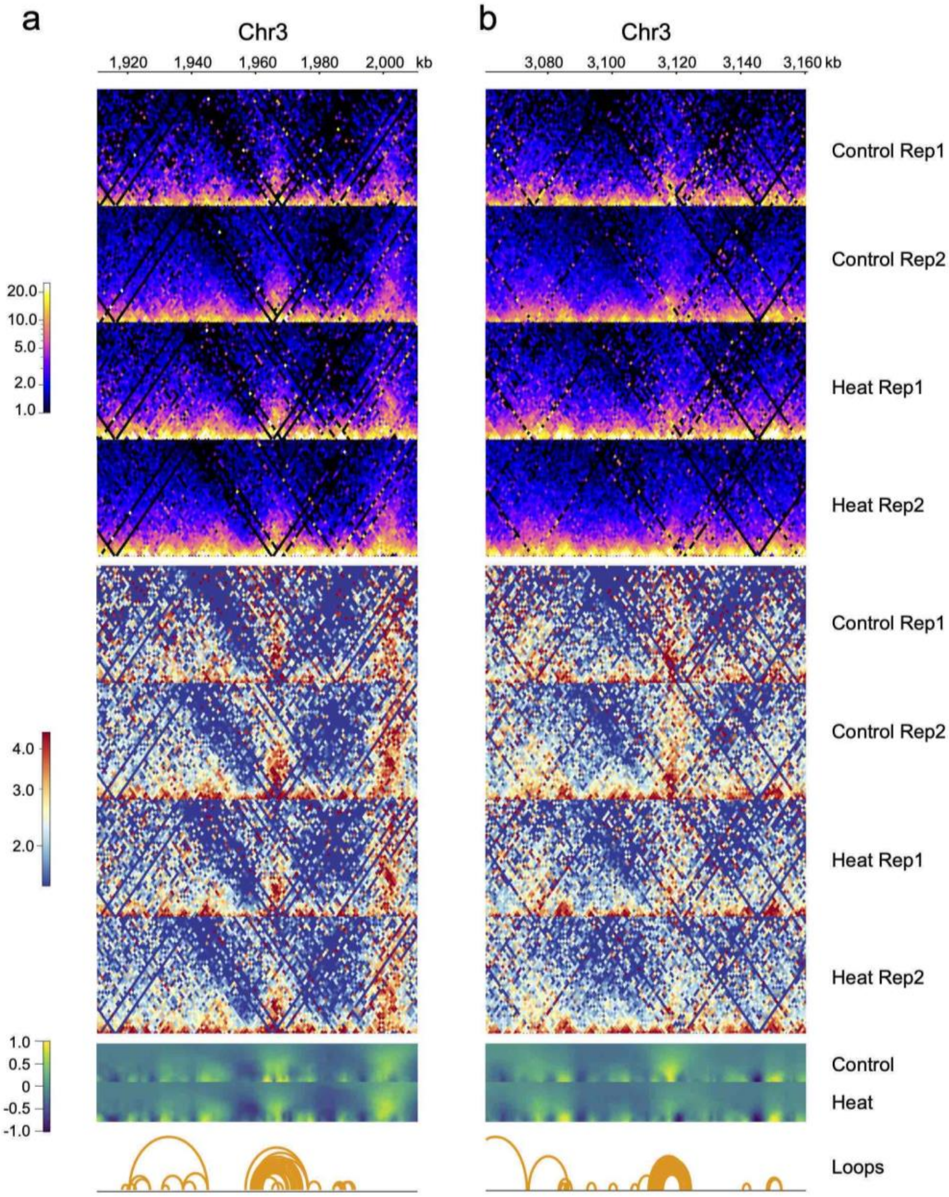
a
b
Chr3
1,920
1,940
1,960
1,980
2,000
kb
Chr3
3,080
3,100
3,120
3,140
3,160
kb
Control Rep1
Control Rep2
Heat Rep1
Heat Rep2
20.0
10.0
5.0
2.0
1.0
Control Rep1
Control Rep2
Heat Rep1
Heat Rep2
4.0
3.0
2.0
1.0
0.5
0
-0.5
-1.0
Control
Heat
Loops

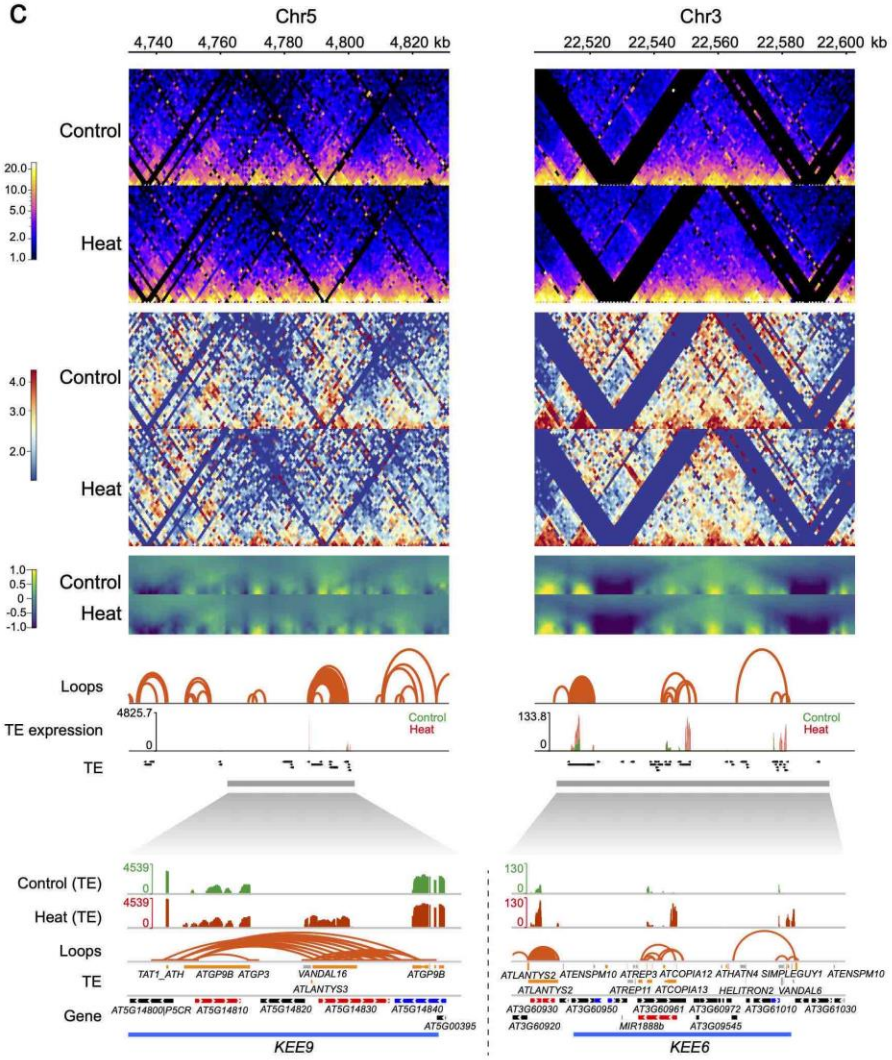

C
Chr5
4,740 4,760 4,780 4,800 4,820 kb
Chr3
22,520 22,540 22,560 22,580 22,600 kb
Control
Heat
20.0
10.0
5.0
2.0
1.0
4.0
3.0
2.0
1.0
0.5
0
-0.5
-1.0
Loops
TE expression
4825.7
0
133.8
Control
Heat
TE
Control (TE)
Heat (TE)
4539
130
Gene
TAT1_ATH ATGP9B ATGP3 VANDAL16 ATGP9B
ATLANTYS3
AT5G14800|P5CR AT5G14810 AT5G14820 AT5G14830 AT5G14840
AT5G00395
KEE9
ATLANTYS2 ATENSPM10 ATREP3 ATCOPIA12 ATHATN4 SIMPLEGUY1 ATENSPM10
ATLANTYS2 ATREP11 ATCOPIA13 HELITRON2 VANDAL6
AT3G60930 AT3G60950 AT3G60961 AT3G60972 AT3G61010 AT3G61030
AT3G60920 MIR1888b AT3G09545
KEE6

d

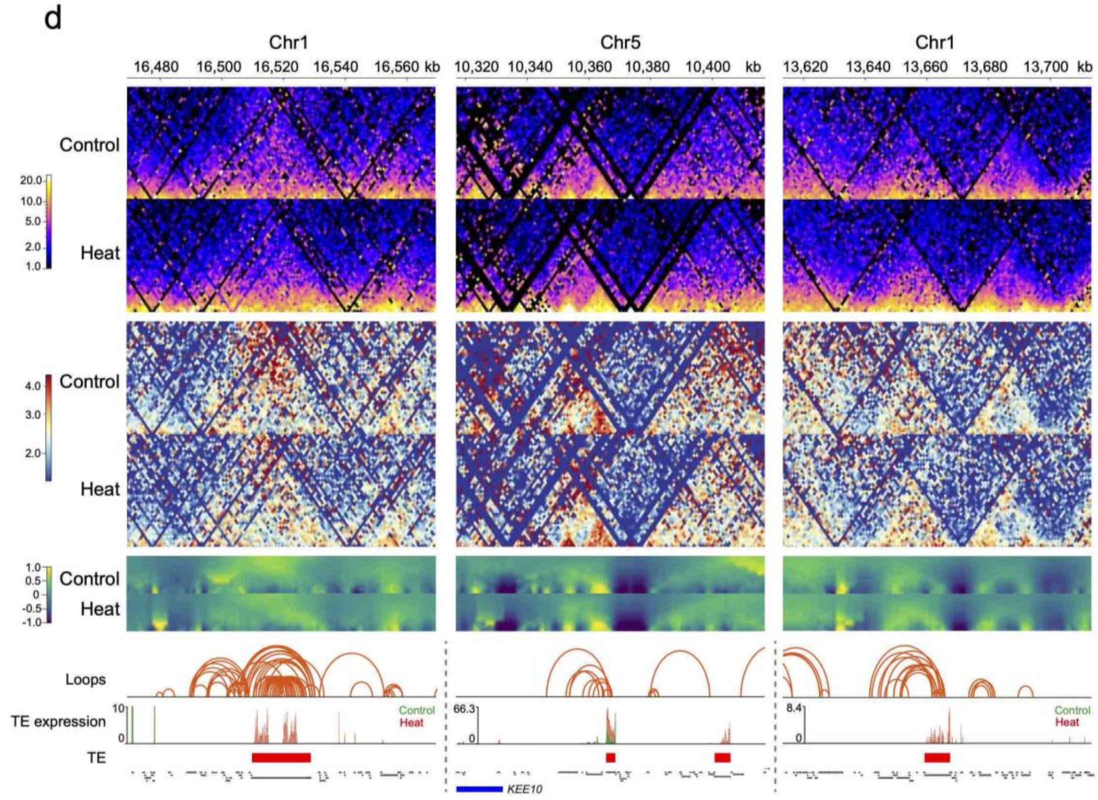

Chr1
16,480 16,500 16,520 16,540 16,560 kb
Chr5
10,320 10,340 10,360 10,380 10,400 kb
Chr1
13,620 13,640 13,660 13,680 13,700 kb
Control
Heat
20.0
10.0
5.0
2.0
1.0
4.0
3.0
2.0
1.0
0.5
0
-0.5
-1.0
Loops
TE expression
10
0
66.3
8.4
Control
Heat
TE
KEE10

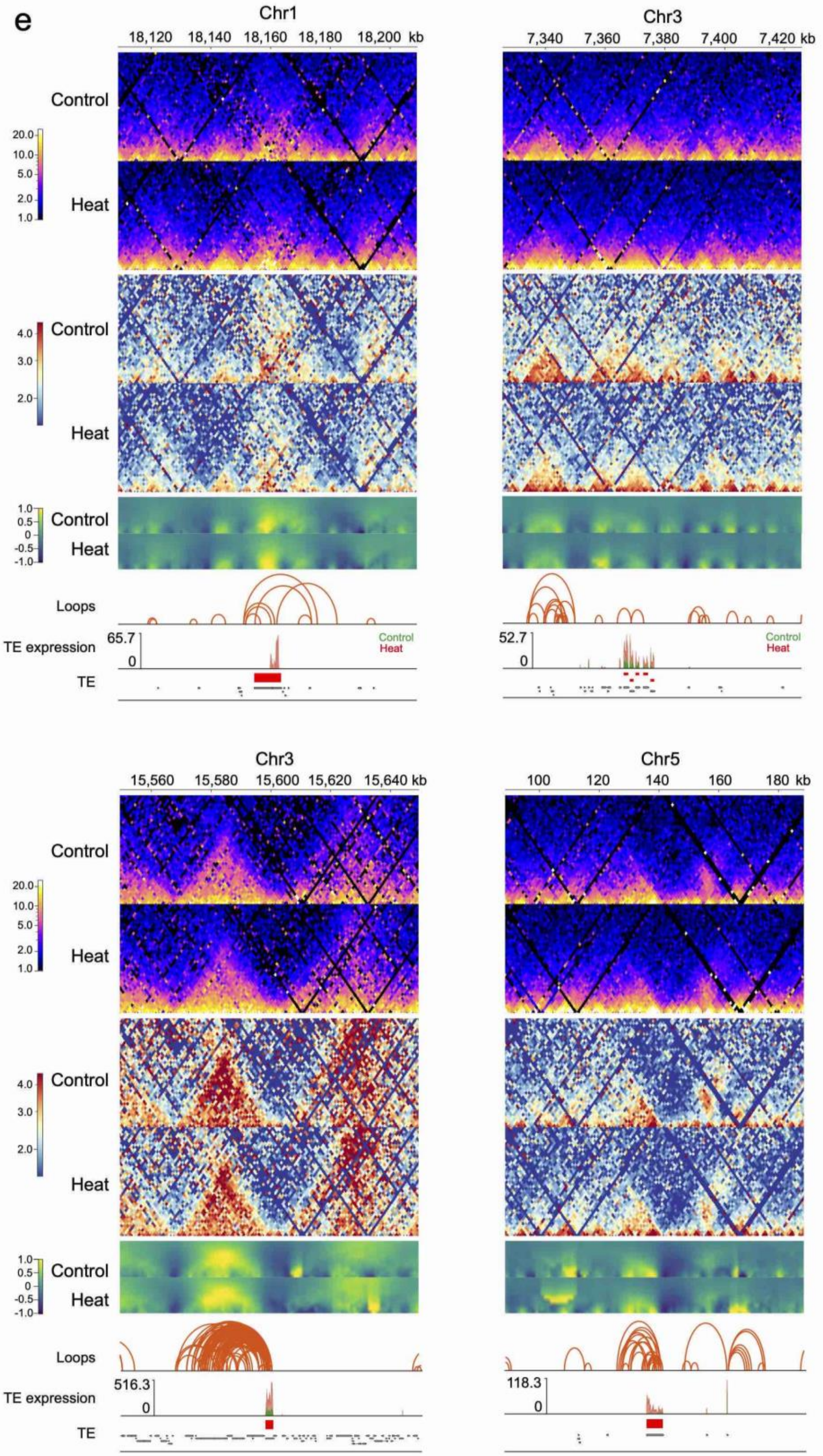

e
Chr1
18,120 18,140 18,160 18,180 18,200 kb
Chr3
7,340 7,360 7,380 7,400 7,420 kb
Control
Heat
20.0
10.0
5.0
2.0
1.0
4.0
3.0
2.0
1.0
0.5
0
-0.5
-1.0
Loops
TE expression
65.7
52.7
0
Control
Heat
TE
Chr3
15,560 15,580 15,600 15,620 15,640 kb
Chr5
100 120 140 160 180 kb
516.3
118.3

Supplementary Figure 14. Changes of local chromatin organization and association with TE expression.

a-b. Changes of local chromatin organization at *KEE3* (a) and *KEE4* (b) loci. Two 100 kb regions (depth: 50 kb) containing *KEE3* (a) and *KEE4* (b), respectively, are shown. The top 4 heatmaps show natural logarithms of 1 kb KR normalized contact frequencies in Control Rep1, Control Rep2, Heat Rep1, and Heat Rep2. The following 4 heatmaps (O/E) show natural logarithms of Observed/Expected contact frequencies in Control Rep1, Control Rep2, Heat Rep1, and Heat Rep2 at 1 kb resolution. The heatmaps at the bottom show TAD-separation scores of different window sizes for each Hi-C genomic bin in Control and Heat. Loops were collected and transformed from a previous study (Methods).

c. Changes of local chromatin organization and TE expression at *KEE9* and *KEE6* loci.

d. Changes of local chromatin organization and TE expression at multiple TE loci from pericentromeric regions.

e. Changes of local chromatin organization and TE expression at multiple TE loci from chromosome arms.

In c-e, 100 kb regions (depth: 50 kb) containing activated TEs are shown. The top 2 heatmaps show natural logarithms of 1 kb KR normalized contact frequencies in Control and Heat. The following 2 heatmaps (O/E) show natural logarithms of Observed/Expected contact frequencies in Control and Heat at 1 kb resolution. The following 2 heatmaps show TAD-separation scores of different window sizes for each Hi-C genomic bin in Control and Heat. Loops were collected and transformed from a previous study (Methods). TE expression track shows the expression level of TEs in Control and Heat. Their expression levels are under the same scale and overlayered together. Only TE expression is shown. The expression of PCGs is not shown (Methods). In c, the expanded panels show TE expression and the corresponding chromatin loops over *KEE9* and *KEE6*. At *KEE9*, RNA-Seq read coverage of Control, Heat, and Recovery is $\log_2$-transformed. In d and e, red blocks indicate locations of heat-activated TEs.
