# Supplementary material for: Heat stress-induced transposon activation correlates with 3D chromatin organization rearrangement in Arabidopsis
Source: Nat Commun. 2020 Apr 20;11:1886. doi: 10.1038/s41467-020-15809-5 (PMC7170881; doi:10.1038/s41467-020-15809-5)
Supplement: Supplementary file 2 — Reporting Summary [file 41467_2020_15809_MOESM2_ESM.pdf]

nature research

Corresponding author(s): Hang He & Weiqiang Qian

Last updated by author(s): Mar 28, 2020

Reporting Summary

Nature Research wishes to improve the reproducibility of the work that we publish. This form provides structure for consistency and transparency in reporting. For further information on Nature Research policies, see Authors & Referees and the Editorial Policy Checklist .

Statistics

For all statistical analyses, confirm that the following items are present in the figure legend, table legend, main text, or Methods section.

n/a	Confirmed	
☐	☒	The exact sample size (*n*) for each experimental group/condition, given as a discrete number and unit of measurement
☐	☒	A statement on whether measurements were taken from distinct samples or whether the same sample was measured repeatedly
☐	☒	The statistical test(s) used AND whether they are one- or two-sided<br>*Only common tests should be described solely by name; describe more complex techniques in the Methods section.*
☒	☐	A description of all covariates tested
☐	☒	A description of any assumptions or corrections, such as tests of normality and adjustment for multiple comparisons
☐	☒	A full description of the statistical parameters including central tendency (e.g. means) or other basic estimates (e.g. regression coefficient) AND variation (e.g. standard deviation) or associated estimates of uncertainty (e.g. confidence intervals)
☐	☒	For null hypothesis testing, the test statistic (e.g. *F*, *t*, *r*) with confidence intervals, effect sizes, degrees of freedom and *P* value noted<br>*Give P values as exact values whenever suitable.*
☒	☐	For Bayesian analysis, information on the choice of priors and Markov chain Monte Carlo settings
☒	☐	For hierarchical and complex designs, identification of the appropriate level for tests and full reporting of outcomes
☒	☐	Estimates of effect sizes (e.g. Cohen's *d*, Pearson's *r*), indicating how they were calculated

Our web collection on statistics for biologists contains articles on many of the points above.

Software and code

Policy information about availability of computer code

Data collection	No software or code were used to collect data.
Data analysis	Fastp (v0.12.2); trim_galore (v0.4.4); TopHat2 (v2.1.1); SAMtools (v1.5); RseQC (v2.6.4); featureCounts (v1.6.1); DESeq2 (v1.14.1); ComplexHeatmap (v1.99.3); R (v3.4.3); gtrellis (v1.6.0); IGV (v2.4.3); HiC-Pro (v2.10.0); bowtie2 (v2.2.9); HiCdat (v0.99.0); HOMER (v4.10); GENOVA (v0.9.98); ggplot2 (3.1.0); agriGO (v2.0); bedops (v2.4.35); bedmap (v2.4.35); coolpup.py (v0.9.3); HiCExplorer (v3.3); pyGenomeTracks (v3.2); seqplots (v1.10.6). All custom scripts have been uploaded into github (https://github.com/Linhua-Sun/Ath_Heat_Hi-C).

For manuscripts utilizing custom algorithms or software that are central to the research but not yet described in published literature, software must be made available to editors/reviewers. We strongly encourage code deposition in a community repository (e.g. GitHub). See the Nature Research guidelines for submitting code & software for further information.

Data

Policy information about availability of data

All manuscripts must include a data availability statement. This statement should provide the following information, where applicable:

- Accession codes, unique identifiers, or web links for publicly available datasets
- A list of figures that have associated raw data
- A description of any restrictions on data availability

Raw Hi-C sequencing reads that support the findings of this study have been submitted to the NCBI Sequence Read Archive under accession number PRJNA545383.
RNA-Seq sequencing reads have been submitted to Gene Expression Omnibus under accession number GSE132415.
The source data underlying Figs. 2b, 2c, 4a,d, 5d, 5e, and Supplementary Figs 1d, 3a, 3b, 4b, 4c, 9d, and 12b.
ChIP-seq datasets using Plant Chromatin State Database (http://systemsbiology.cau.edu.cn/chromstates/).
Genome annotation useing Araport11 (https://www.araport.org).

Reference genome using TAIR10 (https://www.arabidopsis.org).
Used public ChIP-Seq, MNase-Seq, and ChAP-Seq datasets were summarized in Supplementary Data 7.
All other relevant data supporting the key finding of this study are available within the article and its Supplementary Information files or from the corresponding author upon reasonable request.

Field-specific reporting

Please select the one below that is the best fit for your research. If you are not sure, read the appropriate sections before making your selection.

- [x] Life sciences
- [ ] Behavioural & social sciences
- [ ] Ecological, evolutionary & environmental sciences

For a reference copy of the document with all sections, see nature.com/documents/nr-reporting-summary-flat.pdf

Life sciences study design

All studies must disclose on these points even when the disclosure is negative.

Sample size	No sample-size calculations were performed. Sample size was determined to be adequate based on the consistency of measurements.
Data exclusions	No data exclusions.
Replication	Two biological replicates were used for Hi-C and RNA-Seq. The reproducibility of biological replicates were shown by PCA (RNA-Seq) in Supplementary Figure 1b and Hi-C related quality control in Supplementary Figure 5.
Randomization	Biological replicates were randomly set. Each biological replicates consisted of randomly pooled seedlings.
Blinding	Investigators were not blinded to Arabidopsis under different conditions during experiments. Data reported are not subjective.

Reporting for specific materials, systems and methods

We require information from authors about some types of materials, experimental systems and methods used in many studies. Here, indicate whether each material, system or method listed is relevant to your study. If you are not sure if a list item applies to your research, read the appropriate section before selecting a response.

Materials & experimental systems

n/a	Involved in the study	
[ ]	[x]	Antibodies
[x]	[ ]	Eukaryotic cell lines
[x]	[ ]	Palaeontology
[x]	[ ]	Animals and other organisms
[x]	[ ]	Human research participants
[x]	[ ]	Clinical data

Methods

n/a	Involved in the study	
[x]	[ ]	ChIP-seq
[x]	[ ]	Flow cytometry
[x]	[ ]	MRI-based neuroimaging

Antibodies

Antibodies used	Anti-H3K27me1 (Millipore #07-448), and anti-rabbit Alexa Fluor@488 (A23220, Abbkine) was used as the secondary antibody.
Validation	All antibodies used in this study were commercially available. The validation of antibody was performed with WB by the provider using the antigen (see https://www.merckmillipore.com/CN/en/product/Anti-monomethyl-Histone-H3-Lys27-Antibody,MM_NF-07-448 and https://www.abbkine.com/product/dylight-488-goat-anti-rabbit-igg-a23220/).
