# Supplementary material for: Heat stress-induced transposon activation correlates with 3D chromatin organization rearrangement in Arabidopsis
Source: Nat Commun. 2020 Apr 20;11:1886. doi: 10.1038/s41467-020-15809-5 (PMC7170881; doi:10.1038/s41467-020-15809-5)
Supplement: Supplementary file 3 — Description of Additional Supplementary Files [file 41467_2020_15809_MOESM3_ESM.docx]

**Description of Additional Supplementary Files**

Title: Supplementary Data 1

Description: List of differentially expressed genes under heat stress.

Title: Supplementary Data 2

Description: GO results of differentially expressed genes.

Title: Supplementary Data 3

Description: List of differentially expressed TEs under heat stress.

Title: Supplementary Data 4

Description: Summary statistics for Hi-C next-generation sequencing.

Title: Supplementary Data 5

Description: List of heat-activated TEs overlapping with small chromatin loops.

Title: Supplementary Data 6

Description: Primers used in this study.

Title: Supplementary Data 7

Description: Summary of public datasets used in this study.
